# Development of “LvL UP 1.0”: a smartphone-based, conversational agent-delivered holistic lifestyle intervention for the prevention of non-communicable diseases and common mental disorders

**DOI:** 10.3389/fdgth.2023.1039171

**Published:** 2023-05-10

**Authors:** Oscar Castro, Jacqueline Louise Mair, Alicia Salamanca-Sanabria, Aishah Alattas, Roman Keller, Shenglin Zheng, Ahmad Jabir, Xiaowen Lin, Bea Franziska Frese, Chang Siang Lim, Prabhakaran Santhanam, Rob M. van Dam, Josip Car, Jimmy Lee, E Shyong Tai, Elgar Fleisch, Florian von Wangenheim, Lorainne Tudor Car, Falk Müller-Riemenschneider, Tobias Kowatsch

**Affiliations:** ^1^ Future Health Technologies, Singapore-ETH Centre, Campus for Research Excellence and Technological Enterprise (CREATE), Singapore, Singapore; ^2^ Saw Swee Hock School of Public Health, National University of Singapore, Singapore, Singapore; ^3^ Neuroscience and Mental Health, Lee Kong Chian School of Medicine, Nanyang Technological University Singapore, Singapore, Singapore; ^4^ Centre for Digital Health Interventions, Institute of Technology Management, University of St. Gallen, St. Gallen, Switzerland; ^5^ Centre for Digital Health Interventions, Department of Management, Technology, and Economics, ETH Zurich, Zurich, Switzerland; ^6^ Department of Exercise and Nutrition Sciences, Milken Institute School of Public Health, George Washington University, Washington DC, DC, United States; ^7^ Centre for Population Health Sciences, LKCMedicine, Nanyang Technological University, Singapore, Singapore; ^8^ Department of Primary Care and Public Health, School of Public Health, Imperial College London, London, United Kingdom; ^9^ Research Division, Institute of Mental Health, Singapore, Singapore; ^10^ North Region & Department of Psychosis, Institute of Mental Health, Singapore, Singapore; ^11^ Yong Loo Lin School of Medicine, National University of Singapore, Singapore, Singapore; ^12^ Digital Health Center, Berlin Institute of Health, Charite University Medical Centre Berlin, Berlin, Germany; ^13^ Institute for Implementation Science in Health Care, University of Zurich, Zurich, Switzerland; ^14^ School of Medicine, University of St. Gallen, St. Gallen, Switzerland

**Keywords:** chatbot, mHealth, behaviour change, cognitive behavioural therapy, diabetes, depression, health programme

## Abstract

**Background:**

Non-communicable diseases (NCDs) and common mental disorders (CMDs) are the leading causes of death and disability worldwide. Lifestyle interventions *via* mobile apps and conversational agents present themselves as low-cost, scalable solutions to prevent these conditions. This paper describes the rationale for, and development of, “LvL UP 1.0″, a smartphone-based lifestyle intervention aimed at preventing NCDs and CMDs.

**Materials and Methods:**

A multidisciplinary team led the intervention design process of LvL UP 1.0, involving four phases: (i) preliminary research (stakeholder consultations, systematic market reviews), (ii) selecting intervention components and developing the conceptual model, (iii) whiteboarding and prototype design, and (iv) testing and refinement. The Multiphase Optimization Strategy and the UK Medical Research Council framework for developing and evaluating complex interventions were used to guide the intervention development.

**Results:**

Preliminary research highlighted the importance of targeting holistic wellbeing (i.e., both physical and mental health). Accordingly, the first version of LvL UP features a scalable, smartphone-based, and conversational agent-delivered holistic lifestyle intervention built around three pillars: Move More (physical activity), Eat Well (nutrition and healthy eating), and Stress Less (emotional regulation and wellbeing). Intervention components include health literacy and psychoeducational coaching sessions, daily “Life Hacks” (healthy activity suggestions), breathing exercises, and journaling. In addition to the intervention components, formative research also stressed the need to introduce engagement-specific components to maximise uptake and long-term use. LvL UP includes a motivational interviewing and storytelling approach to deliver the coaching sessions, as well as progress feedback and gamification. Offline materials are also offered to allow users access to essential intervention content without needing a mobile device.

**Conclusions:**

The development process of LvL UP 1.0 led to an evidence-based and user-informed smartphone-based intervention aimed at preventing NCDs and CMDs. LvL UP is designed to be a scalable, engaging, prevention-oriented, holistic intervention for adults at risk of NCDs and CMDs. A feasibility study, and subsequent optimisation and randomised-controlled trials are planned to further refine the intervention and establish effectiveness. The development process described here may prove helpful to other intervention developers.

## 1. Introduction

Non-communicable diseases (NCDs) — such as cardiovascular disease, diabetes, or cancer — form the primary causes of death and disability, accounting for more than two-thirds of global mortality (1). Common mental disorders (CMDs) — such as depression or anxiety — often coexist with NCDs and are also highly prevalent worldwide, causing significant health and financial burdens (2–4). Although the treatment regimens for different NCDs and CMDs can differ, disease-specific recommendations have a common feature in that they emphasise the importance of lifestyle behaviours, including physical activity, diet, tobacco smoking, alcohol consumption, sleep, stress and emotional regulation, as vital modifiable risk factors associated with the prevention and management of NCDs (5) and CMDs (6).

Nevertheless, in many regions across the world, urbanisation and economic growth have gone hand in hand with the widespread adoption of modern lifestyles, characterised by diets rich in sugar and saturated fats, low levels of physical activity, and demands on personal resources associated with high levels of stress. Indeed, clustering of lifestyle risk factors has been linked to the high incidence of NCDs (7–10) and CMDs (11) globally, posing a significant threat to healthcare systems in terms of increased socio- and health-economic costs (12, 13). Furthermore, the prevalence of NCDs and CMDs is not always equally distributed. There is a steep socioeconomic gradient for both NCDs and CMDs' incidence in which specific population subgroups, for example those from low socio-economic backgrounds or low educational attainment, are particularly vulnerable (14).

In response to the high levels of NCDs and CMDs, different public health promotion initiatives have been introduced to support nationwide efforts to reduce the prevalence of such diseases and their burden on health services (15). Modifying lifestyle behaviours at the national level is, however, a substantial challenge and traditional face-to-face interventions are neither scalable nor sustainable (16, 17). Digital health interventions (DHIs), on the other hand, have the potential to transform health care by providing effective, low-cost, and scalable support to individuals adopting and maintaining a healthy lifestyle (18).

While DHIs targeting lifestyle behaviours are available and have shown to be effective (19, 20), there are some shortcomings that justify the need to develop new interventions. Firstly, many available DHIs predominantly target single health conditions or health behaviours (21). Given the inextricable links between physical and mental health (2, 4, 22–24), the combined effects of holistic interventions (targeting body and mind) may have a larger total effect than any separate pathway for NCD and CMD prevention. Secondly, most DHIs have been developed in Western countries for Western populations (25, 26). An intervention that is effective in one setting or for one population could be non-effective or even harmful elsewhere (27), thus tailoring and cultural adaptation for the target population are essential. Thirdly, those who are most in need of changing their lifestyle for the prevention of NCDs and CMDs (i.e., people from lower socioeconomic backgrounds) are also the hardest to reach with health interventions (28–31). Therefore, DHI designers must make a concerted effort to reach and engage these hard-to-reach populations.

In summary, if nationwide lifestyle behaviour change is sought, it is crucial to develop DHIs that target physical and mental health holistically, that are tailored to the local context and the target population, and that are appealing, engaging, and accessible to a wide range of people from different backgrounds. This paper describes the rationale and development of “LvL UP 1.0″, a smartphone-based lifestyle intervention that aims to overcome the limitations of previous DHIs by targeting holistic health and wellbeing to prevent NCDs and CMDs.

## 2. Methods

### 2.1. Research team

The development of LvL UP 1.0 was led by a team of interdisciplinary researchers with expertise in digital health, behavioural science, mental health, cultural adaptation of DHIs, computer science, technology assessment, and marketing, and was supervised by the Future Health Technologies' Scientific Advisory Board, an external panel composed of six international experts in the fields of behavioural health, medicine, health engineering, computer science, economics, and social sciences. Feedback from the Scientific Advisory Board was incorporated into the first version of LvL UP and helped reinforce some of the early decisions regarding the interventions' scope and content (e.g., adopting a holistic approach).

### 2.2. Setting and Target Population

The South-East Asia region has the highest reported prevalence of CMDs globally (32), and NCDs are the number one cause of premature death (7). Singapore, a small multi-ethnic country in South-East Asia, has undergone rapid urbanisation in recent decades and its healthcare system is facing challenges arising from the increase of NCDs such as type 2 diabetes and CMDs such as depression (33), their related risk factors (34–37), and their associated socio- and health-economic costs (38, 39).

Singapore provides an ideal setting for the implementation of DHIs given it has one of the highest rates of both smartphone ownership (88%) and daily use (3 h and 12 min on average) in the world (40), coupled with several government initiatives aimed at promoting digital adoption and bridging any potential digital divide such as the Smart Nation Initiative (41) or the Seniors Go Digital programme (42).

Therefore, the LvL UP intervention has been developed to target a multi-ethnic Asian population (largely Chinese, Indian, and Malay) at risk of developing NCDs and CMDs within the Singapore context.

### 2.3. Intervention development frameworks

Two complimentary frameworks were used to guide the development approach: the multiphase optimization strategy (MOST) and the UK Medical Research Council guidelines for developing and evaluating complex interventions. Conceptually rooted in engineering, MOST provides a methodological framework for building, optimising, and evaluating multicomponent interventions (43). The present manuscript reports on the preparation phase of MOST, whereby researchers develop a conceptual model and decide on the structure, mode of delivery, and other key aspects of the intervention. The Medical Research Council guidelines for developing and evaluating complex interventions also underpins the formative research that was conducted as part of the initial stages of intervention development, addressing core elements in intervention design such as context, stakeholder engagement, programme theory, key uncertainties, intervention refinement, and economic considerations (27).

Taken together, these frameworks informed four distinct phases and set of activities which contributed to the development of LvL UP 1.0 (Table 1), including: (i) preliminary research, (ii) selecting intervention components and developing the conceptual model, (iii) whiteboarding to develop a prototype intervention, and (iv) testing and intervention refinement.

**Table 1:** Development process of the LvL UP intervention.

Phase (date)	Activity	Outcome
1. Preliminary research (Q1-Q4 2021)	- Stakeholder engagement (qualitative studies) - Systematic market analyses of existing DHIs	- Understand the unmet needs of the target population and the context in which the intervention will be delivered - Define the proximal and distal outcomes
2. Selecting intervention components and developing the conceptual model (Q3 2021 – Q2 2022)	- Review of scientific evidence and suitable theoretical frameworks (including a review on factors influencing adherence to DHIs and an umbrella review to identify effective behaviour change techniques of DHIs targeting NCDs and CMDs)	- Identify the target behaviours, what needs to change and how to do so (intervention content) - Conceptual model (“driving engine”) of LvL UP
3. Whiteboarding and prototype design (Q4 2021 – Q3 2022)	- Define intervention logic - Follow User Interface (UI) design principles to design application software - Sketches, wireframes and producing intervention content (e.g., coaching session dialogues and life hacks for each pillar)	- Prototype intervention
4. Testing and refinement (Q3 – Q4 2022)	- Pilot testing and hackathons focused on technical aspects and visual content of the app - Intervention refinement	- LvL UP 1.0 intervention

DHIs, Digital health interventions; NCDs, Non-Communicable Diseases; CMDs, Common Mental Disorders.

### 2.4. Phase 1: preliminary research

#### 2.4.1. Stakeholder engagement

Meaningful engagement with key stakeholders is conceived as an integral part of the intervention development process in MOST and the UK Medical Research Council's guidance for developing and evaluating complex interventions (27, 43). Considering LvL UP's aim to prevent NCDs and CMDs *via* lifestyle changes, we conducted qualitative studies with key target populations: potential end-users (young and middle-aged adults) and health supporters. This involved (i) a focus group study with 30 Singaporean young adults (university students), including one-on-one interviews with 11 mental health supporters (faculty members offering pastoral care, a mental health first-aider, counsellors, psychologists, clinical psychologists, and psychiatrists) (44), and (ii) a focus group study with 35 Singaporean middle-aged adults (45).

The qualitative study with university students and mental health supporters recruited participants *via* social media (students) and professional networks (mental health supporters), as well as using snowballing. The focus group study targeting middle-aged adults recruited participants *via* the Facilitators Network Singapore (a local company which assisted with professional focus group facilitation), as well as online advertisements on the Singapore-ETH Centre's website and social media. Specific to this second study, a purposive sampling procedure was used to recruit participants whereby those with indicators of lower socioeconomic status were prioritised (i.e., invited to participate first).

Both studies were conducted online using a videoconferencing platform (Zoom Video Communications, Inc., San Jose, CA) and were analysed using a thematic analysis approach (46, 47) to explore perceptions of, and barriers and facilitators to the use of, DHIs for lifestyle change in Singapore. However, the focus group study targeting young adults had a higher emphasis on mental health (CMD prevention), while the focus group study targeting middle-aged adults put a stronger emphasis on physical health (NCD prevention).

Findings from the two studies highlighted end-users' positive attitude towards DHIs targeting holistic health and wellbeing (both physical and mental health). In addition, participants felt that keeping users engaged long-term represents a significant challenge for any DHI. Factors such as user-friendliness, tailored content and personalisation, use of reminders, government-backing, and gamification were mentioned explicitly as important facilitators to intervention engagement. In terms of mental health perceptions, young adults highlighted that stigma plays an important role in preventing Singaporeans from seeking mental health services and that DHIs might be a potential avenue to access mental health support anonymously. While DHIs were viewed as sufficient to support lifestyle behaviour change, ends-users and mental health supporters emphasised the importance of human support alongside a digital intervention (i.e., a blended approach) to increase mental health treatment effectiveness.

#### 2.4.2. Market analysis of existing DHIs

To explore the market and evaluate the features of currently available DHIs, we conducted two systematic market analyses of the top-funded companies offering DHIs for the prevention, treatment, and management of two of the most common NCDs and CMDs; type 2 diabetes (48) and depression (49). Findings revealed that most DHIs have been developed in Western countries, with the US leading. In addition, few DHIs utilised technology beyond a simple information delivery, communication, or health tracking mechanism (50, 51), for example by incorporating just-in-time adaptive interventions (using sensors to intervene and deliver support at the most opportune moments) or using AI to offer automated support mechanisms such as conversational agents, which limits their scalability. Last, more than half of the companies evaluated did not generate any scientific evidence to support the effectiveness of their intervention.

In addition to the systematic market analyses, we conducted a review of Singapore's landscape regarding DHIs to identify what is currently being implemented in the country that has been designed for the Singapore context. Singapore's Health Promotion Board offers DHIs targeting lifestyle behaviours including physical activity—National Steps Challenge (52)—and healthy eating—Eat, Drink, Shop Healthy Challenge (53)—through its Healthy365 app. These DHIs offer gamification but rely heavily on extrinsic motivation mechanisms, such as monetary incentives and rewards, to drive behaviour change. Another intervention offered in collaboration with the Health Promotion Board (LumiHealth) takes a more holistic approach to health (54) but is only offered to individuals who own an iPhone and Apple Watch therefore likely targets individuals from a higher socioeconomic background. There are also free resource-based websites focused on information giving and signposting to other support services (e.g., HealthHub.sg and mindline.sg), but these fail to provide any personalised support to individuals.

Taken together, findings from Phase 1 highlighted the importance of providing scalable, holistic health support that is culturally adapted and tailored to the target population.

### 2.5. Phase 2: selecting components and developing the conceptual model

During this phase the research team reviewed the scientific evidence and identified suitable theoretical frameworks to inform the selection of intervention components. In addition, a conceptual model was developed to illustrate how these components are expected to interact with each other and bring about change.

#### 2.5.1. Engagement components

As a groundwork to select effective engagement strategies, we conducted a systematic review to identify the factors influencing long-term adherence to DHIs targeting prevention and management of NCDs (55). Consistent with our focus group discussions, results highlighted four intervention-related factors that have positive effects on adherence: personalisation or tailoring of the content of mHealth apps to the individual needs of the user, reminders in the form of individualised push notifications, user-friendly and technically stable app design, and personal support complementary to the digital intervention. Social and gamification features were also identified as drivers of app adherence across several health domains. These findings were carefully considered when developing the intervention's engagement components.

Moreover, we decided to adopt a relatively novel engagement approach in the healthcare domain consisting of storytelling. This approach has been successfully used to deliver health education content in a low-burden, entertaining manner (56). Stories are universal and are present across cultures and times, which suggests that they meet an enduring psychological need (57). From a health communication perspective, storytelling can be more effective than paradigmatic approaches that rely on empirical and scientific data. In everyday life, humans communicate through narratives and telling stories, thus making this mode of communication more relatable and less cognitively burdensome (58–60). This can be explained through the Social Cognitive Theory, which states that individuals can acquire knowledge through observing others, whether it is in-person or digitally, fiction or non-fiction (61, 62).

Relevant to the prevention of NCDs and CMDs, the conflicts that the story's characters face can reflect common barriers to behaviour change that promotes healthy living, and their ways of overcoming these barriers can model healthy physical activity, dietary choices, and/or adaptive stress coping strategies (57). In this way, direct engagement with story-based media can potentially lead to behaviour change by informing, motivating, or guiding viewers (63). Previous systematic reviews have found that promoting health behaviours through storytelling is a promising strategy (64, 65). Our storytelling approach in LvL UP 1.0 involved the production of short, animated video clips by a game development company based in Singapore.

In addition to the use of personalisation, reminders, gamification, and storytelling, we chose conversational agents as the “vehicle” to deliver the health literacy and psychoeducational components of the intervention. Conversational agents—computer systems, or “digital coaches”, that imitate conversations with humans through text or voice—are a novel and rapidly growing approach to digital health which can improve user experience and engagement (66, 67). Conversational agents have been used successfully in the health care domain (68, 69) and recent studies show that users can establish a working alliance with conversational agents (70–72). Working alliance, also known as therapeutic or helping alliance, is a concept that originates from psychotherapeutic settings and refers to the collaborative quality between clients and health professionals (73). Building up a working alliance is robustly linked to patient engagement, retention (74, 75), and treatment outcomes (76–78). Furthermore, conversational agents can be classified as temporary assistants (79) and as peers with characteristics of high interpersonal closeness. For example, they address participants by their nickname and have a more ordinary look (80).

There are different types of interaction styles that conversational agents can employ (e.g., collaborative, directive, empathetic, pragmatic). A rich literature exists on individual, person-centred, counselling approaches that have been shown to be effective in helping people change health-related behaviours (81). Motivational interviewing is a collaborative, goal-oriented style of communication that elicits behaviour change by helping clients to explore and resolve ambivalence (82). Based on the Self-determination Theory (83), motivational interviewing places special emphasis on the vocabulary for change used during the individual sessions (i.e., the style of delivery), recommending the use of non-controlling language which supports people's autonomy and reflects acceptance, partnership, and compassion.

We decided to frame the communication style of our conversational agent on motivational interviewing, as this counselling approach has been successfully applied across a broad range of settings, populations, languages, formats (e.g., individual, group, digital) and health topics, including fitness, nutrition, medication adherence, and substance use (84). In addition, the coaching sessions include different motivational interviewing-based strategies such as rephasing, using affirmations, and exploring reasons for change. Self-determination Theory also inspired other design decisions for the intervention. For example, we attempted to provide users with the maximum degree of freedom possible when navigating and using the different intervention components. While different recommendations are made throughout the intervention, users always have the option to follow their own path and decide which intervention components to complete and when to do so.

#### 2.5.2. Lifestyle components

Alongside the findings of Phase 1, we conducted an umbrella review of systematic reviews to establish the effectiveness of DHIs targeting NCDs and identify effective behaviour change techniques (BCTs) within these interventions (85). The review was conducted in accordance with the registered protocol (Open Science Framework Registry; 10.17605/OSF.IO/GE2RS) and the Preferred Reporting Items for Systematic Reviews and Meta-Analyses (PRISMA) 2020 statement (86).

Sixty-one articles, spanning 10 health domains and comprising over half a million individual participants, were included in the review. Results indicated that DHIs are favourably associated with improved health outcomes for patients with cardiovascular disease, cancer, type 2 diabetes, asthma, depression, and anxiety. Furthermore, DHIs are effective in improving health-related behaviours and outcomes including physical activity, sedentary behaviour, diet, weight management, medication adherence, and abstinence from substance use in both general and clinical populations. Many interventions reviewed focused on changing lifestyle behaviours, notably diet and physical activity, which might be expected given the overwhelming evidence for diet and physical activity interventions in addressing cardiovascular disease risk factors (87) and preventing diseases (88), including type 2 diabetes (89) and depression (90, 91).

Regarding effective BCTs, there was strong evidence to suggest education, communication with a professional, tailored reminders, goals and planning, feedback and monitoring, and personalization components increase the effectiveness of DHIs targeting NCDs and lifestyle behaviours. Support from a professional, tailored reminders, and cognitive behavioural therapy (CBT) techniques were found to be effective in interventions for CMDs. The full results from the umbrella review will be presented in a separate paper.

In summary, the prevention of NCDs and CMDs requires a holistic intervention paradigm, in which health-promoting behaviour, mental health, and wellbeing are central. In particular, a balanced diet and regular physical activity, alongside CBT for anxiety and stress management, appears to be effective in reducing the risk of developing NCDs and CMDs. These lifestyle components form the focus of the LvL UP 1.0 intervention.

##### 2.5.2.1. Lifestyle components targeting NCDs

The Behaviour Change Wheel (BCW), a theory-driven framework that provides a systematic way of developing interventions (92, 93), was used to identify the BCTs needed to improve physical activity and dietary behaviours. Using the Capability, Opportunity, Motivation, Behaviour (COM-B) model we conducted a behavioural diagnosis based on findings from our Phase 1 preliminary research, the umbrella review on effective BCTs in DHIs targeting NCDs, published literature, and weekly discussions among members of the research team, which helped identify a list of “promising” BCTs. We used the APEASE criteria (Affordability, Practicability, Effectiveness and cost-effectiveness, Acceptability, Side-effects and safety, Equity) to narrow down the list of potential BCTs and select those most suitable for our intervention and local context.

Given the wealth of evidence on diet and physical activity interventions for NCD prevention, we decided to incorporate (and culturally adapt) components from existing evidence-based interventions that have been shown to be effective in improving health outcomes *via* lifestyle change, with a particular focus on culturally tailored programmes whenever possible rather than create an entirely new intervention. Accordingly, the US National Diabetes Prevention Program (DPP) was used as a template to develop the Singapore-specific physical activity and diet intervention content. The DPP features a structured, research-based lifestyle change programme which has been shown to reduce the risk of developing type 2 diabetes by 58% in people with prediabetes (94). The Singapore Physical Activity Guidelines (95) and the Singapore Health Promotion Board's My Healthy Plate and Healthy Hawker Hacks programmes (53, 96) complemented the DPP and contributed to the cultural adaptation of the intervention content.

##### 2.5.2.2. Lifestyle components targeting CMDs

Three leading psychological approaches were used to underpin the mental health intervention components: (i) transdiagnostic cognitive behavioural therapy (CBT), (ii) behavioural activation therapy (BAT), and (iii) positive psychology (PP). These approaches have been used successfully in previous DHIs for CMDs (Andersson et al., 2019) as well as in clinical practice by the mental health experts within the research team. Furthermore, interviews with mental health supporters in Singapore highlighted the importance of working with a transdiagnostic CBT approach, including BAT and PP interventions.

Transdiagnostic CBT is characterised by a focus on cognitive, behavioural, and emotional processes that are shared or common across anxiety and depression, and the adoption of a convergent or integrative scientific approach to address them simultaneously (97). Emotional regulation is a common therapeutic ingredient in the transdiagnostic approach, focused on reducing the experiential and behavioural aspects of negative emotions such as anxiety, depression, and stress (98). In addition, the transdiagnostic CBT approach recommends activities for emotional regulation such as journaling, which offers insight into emotions, identifies emotional and behavioural patterns, and provides relief from emotional distress (99). In the same way, slow-paced breathing exercises serve as an arousal and relaxation mechanism in the process of regulating emotions (100). Transdiagnostic CBT has been used effectively in DHIs to reduce symptoms of anxiety and depression (101–103).

BAT focuses on re-engaging individuals in their usual routines through a goal-oriented approach, in which individuals engage in enjoyable activities (to reconnect with positive reinforcement sources) while reducing escape and avoidance behaviours (to decrease potential sources of negative reinforcement) (99). BAT has been used widely in DHIs (104–106) and is recommended as an active ingredient of interventions addressing the prevention and treatment of CMDs (107–109).

Positive psychology (PP) interventions are considered as a complementary strategy in mental health promotion and treatment. PP involves the identification, development, and evaluation of interventions that aim to enhance wellbeing (110). Studies have demonstrated the efficacy of PP interventions such as expressing gratitude (111) and using personal strengths to enhance well-being and, potentially, to alleviate anxious and depressive symptoms (112). Several self-help DHIs use the PP approach and support its effectiveness (113).

#### 2.5.3. Conceptual model

**Figure 1:**
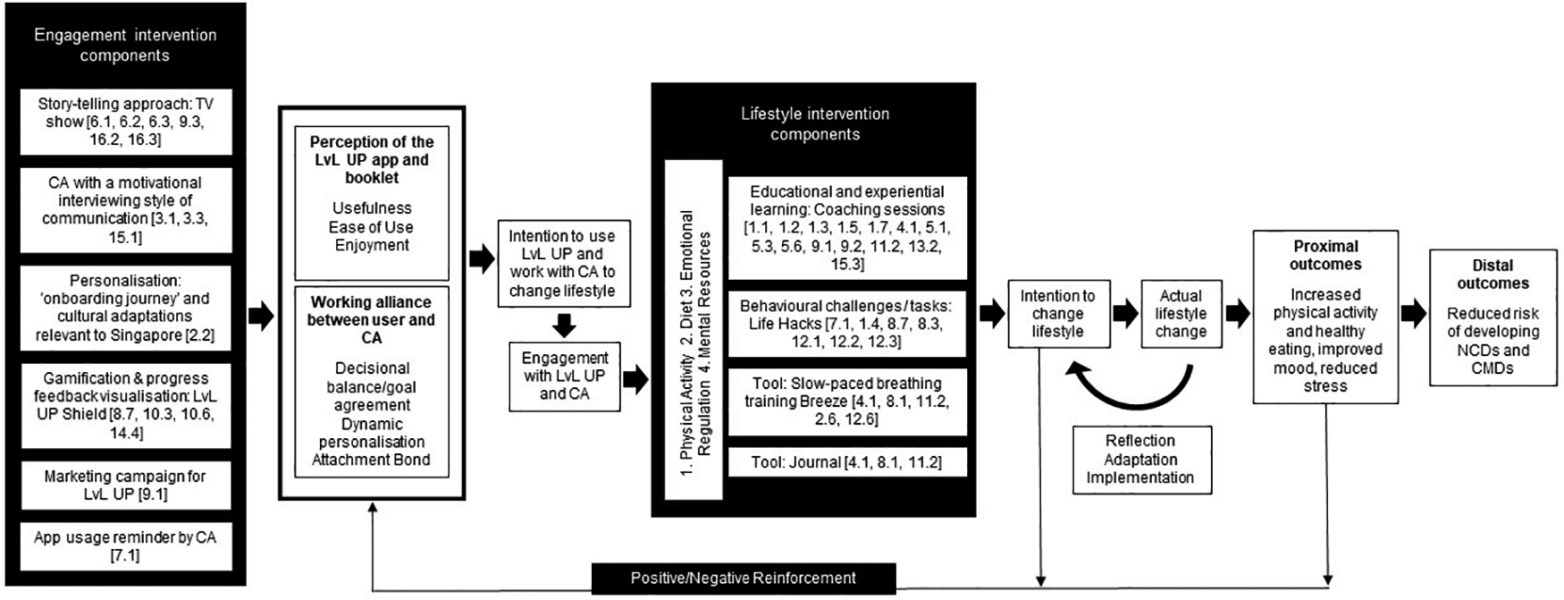
Conceptual model of LvL UP. Black boxes are intervention components. White boxes are intervention outcomes. Conversational Agent (CA), Non-communicable diseases (NCDs), common mental disorders (CMDs). ‘[No.]' refers to the behaviour change technique labels (93): 1.1. Goal setting (behaviour), 1.2. Problem solving, 1.3. Goal setting (outcome), 1.4. Action planning, 1.5. Review behaviour goal(s), 1.7. Review outcome goal(s), 2.2. Feedback on behaviour, 2.6. Biofeedback, 3.1. Social support (unspecified), 3.3. Social support (emotional), 4.1. Instruction on how to perform the behaviour, 5.1. Information about health consequences, 5.3. Information about social and environmental consequences, 5.6. Information about emotional consequences, 6.1. Demonstration of the behaviour, 6.2. Social comparison, 6.3. Information about others’ approval, 7.1. Prompts/cues, 8.1. Behavioural practice/rehearsal, 8.3. Habit formation, 8.7. Graded tasks, 9.1. Credible source, 9.2. Pros and cons, 9.3. Comparative imagining of future outcomes, 10.3. Non-specific reward, 10.6. Non-specific incentive, 11.2. Reduce negative emotions, 12.1. Restructuring the physical environment, 12.2. Restructuring the social environment, 12.3. Avoidance/reducing exposure to cues for the behaviour, 12.6. Body changes, 13.2. Framing/reframing, 14.4. Reward approximation, 15.1. Verbal persuasion about capability, 15.3. Focus on past success, 16.2. Imaginary reward, 16.3. Vicarious consequences.

Figure 1 outlines the conceptual model of the LvL UP 1.0 intervention. The model reflects the causal chain triggered by intervention components that target (i) engagement of LvL UP users and (ii) the actual lifestyle outcomes of interest for the prevention of NCDs and CMDs. Based on the Theory of Planned Behavior (114), technology acceptance (115), and working alliance research (16, 70) we envision that once positive evaluations of the app usage experience and working alliance with the conversational agent are created, users will intend to use the app, exhibiting a state of engagement. Once engaged, individuals are then able to benefit from the lifestyle intervention components, which in turn empowers users to set behavioural intentions and incorporate the “actual behaviours” in their daily lives during a period of reflection, implementation, and experiential learning, as part of the coaching process (116). As individuals change their lifestyle, the proximal outcomes are expected to improve, leading to a positive reinforcement loop *via* greater engagement with the LvL UP 1.0 intervention and, in the long term, reducing the risk of developing NCDs and CMDs. The terms proximal and distal outcomes are used in MOST to highlight the distinction between immediate targets / consequences of the intervention vs. the longer-term, broad-level outcomes that follow such change.

### 2.6. Phase 3: whiteboarding and prototype design

To combine the selected components in phase 2 and develop the first prototype we created sketches and wireframes depicting the intervention logic, when and how the main content is delivered, and a basic design structure. We used MURAL (https://www.mural.co)—a digital whiteboard tool that enables teams to collaborate visually and brainstorm—to facilitate all whiteboarding activities. This allowed us to visualise and discuss different alternatives before developing the actual prototype.

Once the team agreed that the sketches and wireframesreflected and appropriately translated the underpinning research and formative activities of LvL UP 1.0 (phases 1 and 2), the software development team used MobileCoach for the technical implementation. MobileCoach (www.mobile-coach.eu) is an open-source software platform for digital biomarker and intervention research (117, 118). MobileCoach has been successfully used for the development of a digital biomarker for asthma control (119–121), the delivery of digital health literacy (72), physical activity (122), personality development (123), chronic pain (68), childhood obesity (124) and holistic wellbeing during the COVID-19 pandemic (125).

MobileCoach is a client-server system. On the MobileCoach server, intervention content and logic are defined. The MobileCoach client implements a talk and tool paradigm to deliver interventions and collect relevant intervention data from study participants (126). The “talk” is implemented as a conversational agent that imitates the conversation with a human being (79). The “tools” aim to encourage users to use the app and apply and practice what has been “learned” from the conversational agent. The MobileCoach client application is available to download on smartphones running iOS or Android. All communication between the clients and the server are encrypted.

### 2.7. Phase 4: testing and refinement

Usability is one of the main barriers to the adoption of DHIs (127). Digital tools are likely to be rejected by users if they have usability problems (128–130) and, specific to DHIs developed as part of research projects, it is crucial that the app matches the sophistication that users expect from “real-world” apps (131). For all this, we decided to incorporate app testing early in the development process. Inspired by agile development methodology, we took an iterative approach to the development of the LvL UP prototype whereby iterations developed by the software team were regularly reviewed and tested in internal “hackathons” by the research team. An opportunistic sample of 10 users (i.e., not part of the LvL UP development team but working in the same institution and recruited *via* the Future Health Technologies' internal mailing list and personal networks) also contributed to the hackathons by systematically testing the app and providing feedback for refinement. Testing was primarily aimed at identifying bugs and ensuring the app is stable from a technical point of view. In addition, this stream of work resulted in minor content- (e.g., correcting typos, shorting up text) and aesthetic-related changes in the app (e.g., changing colours, repositioning some elements).

## 3. LvL UP 1.0 intervention

Following stages 1 to 4 we developed the first version of LvL UP, which features a smartphone-based and conversational agent-delivered holistic lifestyle intervention built around three pillars: Move More (focused on physical activity), Eat Well (focused on nutrition and healthy eating), and Stress Less (focused on emotional regulation and wellbeing). The different lifestyle and engagement components forming the LvL UP 1.0 intervention and resulting from the development process are described below, with a high-level overview of relevant BCTs provided as part of the conceptual model (Figure 1). In addition, The TIDieR (Template for Intervention Description and Replication) checklist is available as an online Supplementary Material (File S1). A diagram showing the intervention flow is also available as an online Supplementary Material (File S2), as well as a selection of screenshots from the LvL UP 1.0′s app (File 3).

### 3.1. Engagement components

#### 3.1.1. Storytelling approach

The intervention content and components that form the LvL UP 1.0 intervention are framed within an overarching story of four characters– Kai, Alex, Dana, and Ray—that portray typical Singaporean lifestyles. Over the course of LvL UP, users follow Kai, Alex, Dana, and Ray as they struggle to balance their work and personal life. Each of the characters face challenges in the areas of physical activity, diet, and mental health in different ways. As users progress through the intervention, they find out more about how these characters overcome their respective challenges to become the best versions of themselves, both physically and mentally. This overarching story is presented to users in the form of four animated video clips, which are unlocked as users complete levels. We refer to these four videos as the four episodes of LvL UP Season 1.

In addition to the overarching story, during onboarding users choose one out of the four characters as the conversational agent that they communicate with throughout the intervention. The main mode of communication between the user and the chosen conversational agent is through the coaching sessions, within which the characters share “mini-stories” with the user. Mini-stories are short segments of dialogue where the conversational agent shares their personal experiences and struggles related to the specific coaching session that is being completed. Therefore, users will receive a different set of mini-stories depending on which character they choose as their conversational agent. An example of the different mini-stories that are presented for each character, and a detailed description of the overarching story, is available as an online Supplementary Material (File S2).

#### 3.1.2. LvL Up shield

The LvL UP shield is the main visualisation of progress through the intervention Supplementary Material (File S3). It comprises four different sections, each representing the four different categories of tasks that users must do in order to complete a level: LvL UP Coaching sessions, LvL UP Life Hacks, LvL UP Tools, and LvL UP Basics (see next section on lifestyle intervention components). LvL UP Basics includes the intermediary dialogues, as well as research questionnaires that are used to evaluate LvL UP 1.0. There are three different variations of the shield for each of the three levels. The shield is separated into “shield pieces” that the users are awarded incrementally upon the completion of each task. Each level comprises of 14 shield pieces, with a different criterion to collect these 14 shield pieces in each level. An overview of the shield logic is available as an online Supplementary Material (File S2).

#### 3.1.3. LvL Up notifications

The LvL UP 1.0 logic includes different notifications to foster engagement with the app and its different intervention components. Coaching sessions auto-start at the appointment time pre-selected by the participant. The first lines of the dialogue are sent as push notifications to the participant's smartphone (if the participant does not have the LvL UP app already open). In addition, participants receive a push notification each day regarding their daily suggested Life Hack. This notification may be delivered around morning (6–8am), lunch (11am-1pm), or dinner time (5–7pm), depending on the specific life hack. If the user agrees to try the suggested Life Hack, or chooses a different Life Hack from the library of options, a follow up notification will be delivered later in the day asking the participant (i) if they completed the Life Hack and (ii) to provide a usefulness rating out of 5 stars.

Engagement reminders are triggered by the conversational agent as in-app notifications if a user starts, but does not complete, a coaching session (after 1 h, 1 day, and 3 days of no interaction) and through a separate communication channel (SMS/WhatsApp/email) (after 5 days and after 7 days) to positively influence the intention of the participant to continue working with the CA. A detailed overview of the different notification mechanisms is available as an online Supplementary Material (File S2).

### 3.2. Lifestyle components

#### 3.2.1. LvL Up coaching sessions

Coaching sessions are text-based, motivational interview-inspired dialogues between the user and the conversational agent focused on one of the three pillars (i.e., Move More, Eat Well, or Stress Less). All interactions between the user and the conversational agent are rule-based, meaning that the routes and content of the conversation are predefined by the research team. There are six coaching sessions within each pillar that are delivered to users in a predefined order from session 1 to session 6. A brief description of each of the sessions within each pillar is available as an online Supplementary Material (File S2).

The length of coaching sessions ranges from 5 to 15 min depending on the content as well as the level of depth the user wishes to go into. Infographics and animated GIFs are interspersed throughout the coaching sessions to break up large chunks of text and condense key points into a more visual format. The default coaching session is the shortest possible path; however, users can select specific option that allow them to receive more detail on a topic if desired. This approach caters to users with different needs and levels of prior knowledge. Moreover, the coaching content is culturally adapted to Singapore where possible. For example, an adapted version of the US CDC's National Diabetes Prevention Program was used to develop the “eat well” pillar, with reference to Asian diet, and the content of the “move more” pillar considers the tropical climate in Singapore when making activity recommendations. All coaching sessions end with the user setting a “behavioural intention” (i.e., a short task that aims to solidify the learnings from the coaching session). The conversational agent asks the user to choose from three options of behavioural intentions and at the beginning of the subsequent coaching session the conversational agent asks the user if they have completed the behavioural intention. Apart from agreeing on a behavioural intention, users also set an appointment with the conversational agent for when they would like to do the next coaching session. An example of the structure of a coaching session is available as an online Supplementary Material (File S2).

In addition, LvL UP 1.0 includes four intermediary dialogues between the user and their chosen conversational agent (on the same chat interface as the coaching session). These are:

(i) Welcome Dialogue: This dialogue introduces users to LvL UP, the three pillars, and the different tasks they have to do to progress throughout the intervention. The dialogue includes a needs assessment where users answer a set of questions on their current mental health status *via* the Patient Health Questionnaire-4 (132); physical activity levels *via* the International Physical Activity Questionnaire—Short Form (133); and nutritional intake *via* the Modified Food Frequency Questionnaire based on My Healthy Plate (53). The responses to this needs assessment determine the order of pillars that the user is recommended to complete.
(ii) Check-In Dialogues: These are short dialogues that occur after a user has finished all tasks within a level. They aim to check-in with the user on how they are finding LvL UP, reflect their accomplishments, and motivate them to continue with subsequent levels. These check-in dialogues are the last tasks that users have to complete at the end of Level 1 and Level 2.
(iii) Thank You Dialogue: This dialogue completes the third and final level of LvL UP. It includes the same needs assessment that users completed on the Welcome Day. The difference in scores between the two assessments is calculated and reflected back to users to communicate their progress. On completion of the Thank You Dialogue, the final episode of the overarching story is unlocked and the user is free to use the tools, life hacks, and repeat coaching sessions as they wish.

#### 3.2.2. LvL Up life hacks

Life hacks are actionable health and wellbeing-related tips that can be easily implemented into the daily routine. They build on the premise that taking very small concrete actions can build upon each other and lead to noticeable changes over time. The bite-sized nature of these activities is intentionally designed to promote self-efficacy and perceived mastery (134, 135). The app contains a library of 48 life hacks (16 per pillar). Users are encouraged to complete one life hack per day. Every day, a specific life hack from the library is suggested that coincides with the pillar of the coaching session that the user is currently completing. Similar to the coaching sessions, a variety of English spoken in Singapore that incorporates elements of Chinese and Malay (i.e., Singlish) was used within Life Hacks where possible and relevant. An overview of all life hacks is available as an online Supplementary Material (File S2).

#### 3.2.3. LvL Up tools: breeze and journal

Breeze and Journal make up the LvL UP tools, which are activities that users can do regularly to complement their learnings from the coaching sessions and life hacks Supplementary Material (File S3). Combining these different types of interactions between the user and the LvL UP 1.0 app is in accordance with a Talk-and-Tools paradigm (126). In addition, the transdiagnostic CBT approach supports the use of journaling and breathing exercises. Breeze is a gamified slow-paced breathing training (136–138). Users are instructed to inhale and exhale at set ratios, and the inhalations and exhalations are detected by the smartphone's microphone. Exhalations push a boat forward along a river. Journal is an online journaling tool where users are given a blank “lined-paper” template as a space for them to freely express their thoughts, feelings, and emotions. To promote emotional literacy (139), users also have the option to track their mood as part of the journal entry by selecting one of four core emotions—happy, sad, anxious, and angry (140)—and an additional layer of more nuanced emotions (e.g., joyful, excited, or content, if users select “happy”). Saved journal entries can be edited or deleted. A detailed description of breeze and journal is also included as part of the coaching dialogues within the stress less pillar, which is intended to support CBT techniques. In line with Self Determination Theory (141), while recommendations are given for the frequency and duration of use of these tools for optimal results, users have the flexibility to decide when and how they would like to use them to fit their personal needs.

#### 3.2.4. Offline resources: LvL Up booklet

In light of concerns surrounding technostress and the attention economy (142), the LvL UP booklet acts as a complementary offline resource, providing users with the option to perform the activities available in the LvL UP 1.0 app without needing a digital device. For Life Hacks, this is in the form of pop out cards with the title and icon of the life hack in the front and a short description of the Life Hack at the back. For Breeze, this is a step-by-step set of instructions to practicing slow-paced breathing with pictures to aid understanding. For Journal, this is a set of example journal entries for inspiration and pages of blank journaling templates where users can choose to write what they wish. Users are encouraged to use both the app and the booklet to get the full LvL UP experience. A digital version of the LvL UP booklet is available as an online Supplementary Material (File S4).

### 3.3. Intended use for LvL UP

LvL UP 1.0 is conceptualised as a smartphone-based intervention with an intensive phase tied to the delivery daily coaching sessions followed by a maintenance phase during which users can continue to interact with the other app features (i.e., tools and Life Hacks). Theoretically, if users complete all coaching sessions in the suggested manner, the intensive intervention phase lasts approximately three to four weeks and the maintenance phase is indefinite.

## 4. Discussion

This paper describes the development of LvL UP 1.0, a smartphone-based and conversational agent–delivered holistic lifestyle intervention that aims to help adults living in Singapore prevent NCDs and CMDs by promoting healthy nutrition, physical activity, and psychological well-being. LvL UP 1.0′s development was driven by a multidisciplinary team and drew upon preliminary research, theoretical frameworks, stakeholder engagement, systematic reviews, market analyses, and usability testing. While stakeholder engagement and market analyses were particularly useful to inform the broad decisions regarding the intervention's scope, existing evidence and theoretical frameworks helped identify the most promising intervention content, with subsequent adaptations to the Singaporean context. Documenting the intervention development phase allows for the opening of “the black box” of the methods, processes, and resulting decisions occurring during intervention design (143), which might help to achieve a greater understanding of intervention effects, as well as informing the development of future interventions.

A key characteristic of the resulting LvL UP 1.0 intervention is its holistic approach to health and wellbeing. This is different from existing DHIs in Singapore and other countries, which typically focus on single health behaviours (e.g., physical activity in the National Steps Challenge) (52), and represents both an innovative intervention approach and research opportunity in the field of digital health. Even with health promotion programmes that target different domains, there is generally a greater emphasis on combining diet, physical activity, and sleep, while other less tangible elements related to mental health and wellbeing (e.g., emotions, life values, social relationships) are hardly integrated (144, 145). This conflicts with contemporary views on mental health, which emphasise the care of both mind and body, highlighting the significance of the whole human entity and the interdependence of its parts: physical, emotional, and spiritual (146).

To the best of our knowledge, no other DHI currently exists in Asia which has similar targets and underlying philosophy as LvL UP (i.e., holistic, prevention-oriented, and with a concerted effort to be accessible and “engaging enough” for traditionally hard-to-reach populations). In addition, LvL UP's emphasis on prevention of NCDs and CMDs *via* lifestyle change fits well with the Ministry of Health's recently released “healthier SG strategy” (147), which aims to redouble efforts to promote healthier lifestyles, improve support for mental health, and has a strong focus on preventive care. LumiHealth, an app designed by Singapore's Health Promotion Board in conjunction with Apple and Evidation Health to encourage the adoption of healthy habits, is somewhat similar to LvL UP in the sense that also features a health programme targeting different facets of an individual's lifestyle, including physical activity, nutrition, mindfulness, and sleep (54). However, it is worth noting that taking part in LumiHealth requires an iPhone and an Apple watch, which are not covered by the programme, and thus may restrict the socioeconomic diversity of the participants. According to recent statistics, only 25% of the population in Singapore uses iOS (148) and there is a well-documented socioeconomic gradient for iPhone use (149).

Another key difference of LvL UP 1.0 from other digital programmes in Singapore (and Asia more broadly) lies with the use of external rewards, which are a cornerstone of LumiHealth and the National Steps Challenge. Incentives in the form of redeemable coins or points are common in the Singaporean app landscape, both for health- and non-health-related apps. The effectiveness of incentives, however, is contested in the behaviour change field (150). While some defend their use as a useful strategy to encourage initial uptake, others argue that they undermine intrinsic motivation, which is thought to be a key process for sustained engagement and constitutes a source of stress for participants (151). Participants of DHIs implementing a reward system in Singapore have also relayed mixed views on incentives (152). Rather than implementing an external reward system, one of the main engagement components in LvL UP consists of storytelling in the form of a short, animated video clips which leverages on intrinsic motivation through immersion (where the user is emotionally invested in the story), fantasy (where the user can use his or her imagination), and fun (where the user can feel a sense of enjoyment) (57, 59).

In addition to promoting self-determined forms of motivation, the use of storytelling is also a promising strategy when it comes to providing a low-burden, entertaining, and scalable intervention which might be appealing to a wide range of population subgroups, including those from lower socio-economic backgrounds. Different experimental studies have shown that storytelling in the form of spoken animations is a highly effective way to communicate complex health information to people with low health literacy, and that information adapted to audiences with low health literacy also suits individuals with high health literacy (153, 154). In addition to the use of storytelling as our primary engagement approach, we also prioritised the recruitment of individuals with low socioeconomic status in our stakeholder engagement to capture the views and experiences from the full socioeconomic spectrum. Inputs from relevant target groups can significantly contribute to the conceptualisation and development of interventions, ensuring the best chance of developing effective digital health solutions (155, 156). To gain additional insights on this topic, we have started a parallel project which aims to better understand how to engage people from disadvantaged socio-economic backgrounds with digital health promotion programmes in Singapore. The results from this project may inform the development of subsequent LvL UP versions as well as its recruitment approach.

In terms of evaluation, once a DHI prototype has been developed, both the MOST and the MRC guidelines highlight the importance of assessing the feasibility of the intervention before progressing to definitive trials (27). Conducting feasibility and pilot studies are helpful for several reasons, such as identifying recruitment or budget problems, adjusting the intervention content and mode of delivery, informing on the accuracy of the measurement tools, and/or estimating the intervention's effect size (157). We are currently conducting a feasibility pilot study with 200 adults in Singapore with the aims of (i) assessing the technical feasibility of the LvL UP 1.0 intervention (i.e., engagement, working alliance with the conversational agent, and technology acceptance), (ii) evaluating recruitment capability for future trials (i.e., effectiveness of different channels and marketing strategies), (iii) evaluating user satisfaction (i.e., experience with features and conversational agent, cultural relevance of content, design preferences), and (iv) exploring preliminary effects of the intervention on the outcomes of interest (i.e., physical activity, diet, mental wellbeing).

Following the feasibility testing of LvL UP 1.0, further refinements building upon the existing lifestyle and engagement components are envisaged before progressing to optimisation and effectiveness trials. We anticipate collecting a wide range of smartphone sensor data to predict the users' states of receptivity to notifications by using a dynamic machine learning model (158, 159), with the goal of delivering LvL UP notifications at the most opportune moments (i.e., creating a just-in-time adaptive intervention) (50, 160). Moreover, while the first version of LvL UP features a fully scalable, self-guided DHI, given the findings from our umbrella review and the recommendations from end-users and mental health supporters in our focus group studies, we intend to introduce dynamic, needs-driven human support. Human support is often demanded by users (161) and has been shown to increase effectiveness and engagement with DHIs (162, 163). Introducing human support, however, greatly affects the intervention's scalability and costs, although this might vary depending on the specific modality of human support that is implemented (e.g., expert vs. peer support). Finally, the intervention content could be expanded to accommodate a wider range of coaching sessions, supporting tools, and topics (e.g., sleep). In summary, the LvL UP intervention is not envisioned as static but rather a malleable intervention, leveraging the potential of digital health to evolve, innovate, and adapt.

Moving forward, the MOST framework includes an optimisation phase in which the performance of the individual intervention components is assessed. This will involve conducting one or more experimental studies (e.g., factorial or micro-randomized trials) that assess the effectiveness of the intervention components (and/or combinations of them), and how they affect each other. Devoting time and effort to optimising the intervention is a critical and often overlooked step before conducting a formal evaluation. Once the intervention is optimised, we will move to the evaluation phase of MOST, which involves assessing the effectiveness of the optimised intervention, for example by means of a randomised controlled trial. This enables researchers to ascertain whether the optimised intervention has a statistically and clinically significant effect on the outcomes of interest.

### 4.1. Strengths and limitation of LvL UP 1.0′s intervention development approach

A strength of the LvL UP 1.0 intervention's development is the systematic use of current evidence, theoretical underpinning, involvement of end users, and market analysis efforts to inform the scope, content, and mode of delivery of the intervention. Further strengths include a multidisciplinary approach facilitated by the research team's varied expertise, including digital health, behavioural science, mental health, cultural adaptation of DHIs, computer science, technology assessment, and marketing. We believe this led to a richer, more comprehensive account of the different alternatives for the first version of LvL UP. Last, the implementation of commonly used and rigorous frameworks such as MOST and the UK MRC framework for developing and evaluating complex interventions also constitutes a key strength as it allowed the research team to incorporate good intervention development practices (e.g., stakeholder consultation or pilot testing) early in the process.

In terms of limitations, while we engaged the end users in the development process by means of two qualitative studies, other relevant stakeholders have not had direct influence in the development of the first prototype (e.g., health officials). We are currently establishing contacts with a wide range of organisations (e.g., the Singapore's Ministry of Health, different university wellbeing offices in Singapore) with the hope that their views and feedback could be applied in future LvL UP versions. In addition, end users were part of our formative studies (phase 1) but have not been engaged in our “whiteboarding” or “testing and refinement” development phases, which could have resulted in meaningful changes ahead of conducting a formal, somewhat large evaluation of the app *via* the feasibility study.

Another limitation comes from the fact that the LvL UP 1.0 intervention started as two separated projects: one focused on NCDs prevention and a second one emphasising the prevention of CMDs. As a result, some of the formative studies (e.g., stakeholder interviews and market analysis) are somewhat focused on their respective targets (i.e., NCDs or CMDs). To gain a better perspective of interventions combining both physical and mental health, we are currently conducting a systematic literature review that aims to (i) provide an overview of holistic DHIs in the general adult population and (ii) examine their effects on related health and behavioural outcomes. This might provide useful information for upcoming LvL UP versions.

## 5. Conclusions

This paper outlines the evidence-based and user-informed development of the LvL UP 1.0 intervention, which aims to help adults living in Singapore prevent the onset of NCDs and CMDs *via* holistic lifestyle changes. LvL UP 1.0 features a novel intervention approach designed to be scalable, engaging, prevention-oriented, holistic (mind and body), and accessible to traditional hard-to-reach populations. The development stages and activities detailed in this paper may help guide future digital intervention development in Asia or elsewhere.

## Data Availability

The original contributions presented in the study are included in the article/**Supplementary Material**, further inquiries can be directed to the corresponding author.

## Supplementary Material

The Supplementary Materials for this article can be found online at: https://www.frontiersin.org/articles/10.3389/fdgth.2023.1039171/full#supplementary-material.
