# Supplementary material for: Development of “LvL UP 1.0”: a smartphone-based, conversational agent-delivered holistic lifestyle intervention for the prevention of non-communicable diseases and common mental disorders
Source: Front Digit Health. 2023 May 10;5:1039171. doi: 10.3389/fdgth.2023.1039171 (PMC10207359; doi:10.3389/fdgth.2023.1039171)
Supplement: Supplementary file 1 [file Datasheet1.zip › Supplementary File 2.DOCX]

**Supplementary File 2**

Figure. LvL UP’s intervention flow.


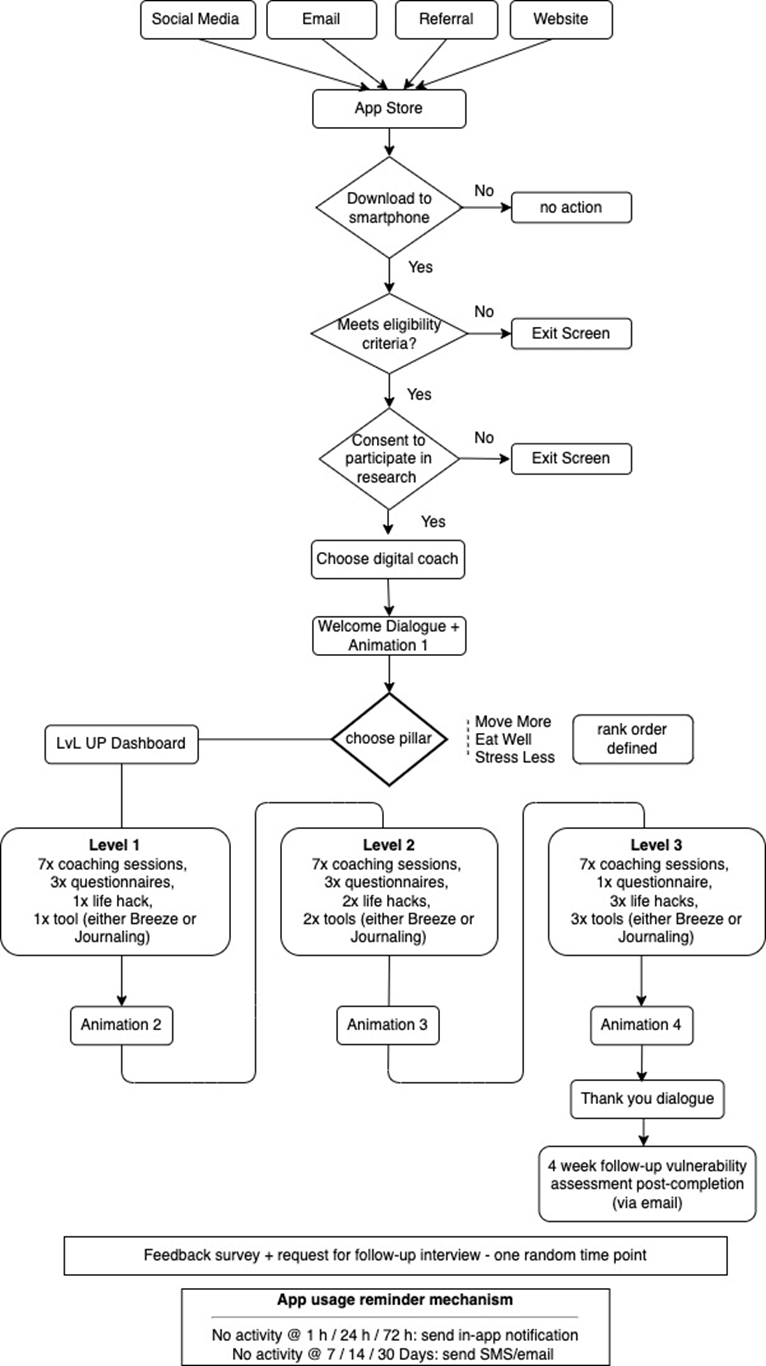


Table. Description of the sessions within each the move more, eat well, and stress less pillars.

|  | Session 1 | Session 2 | Session 3 | Session 4 | Session 5 | Session 6 |
| --- | --- | --- | --- | --- | --- | --- |
| Move More | **Get ready to move!:**   - Why is physical activity important to you? - How can you use your personal strengths to be more active? | **Active body, healthy life**:   - What is physical activity? - Range and types of physical activity - Health benefits of physical activity | **The right physical activity for you:**   - International physical activity guidelines - Setting a physical activity goal - Staying safe while being active | **Jumping over hurdles:**   - Common barriers to being active - Strategies to overcoming different barriers | **Off the couch, on the move:**   - What is sedentary behaviour? - Incorporating positive activity cues - Removing inactivity cues | **Staying on top of physical activity:**   - Move More recap and wrap-up - Physical activity tracking |
| Eat Well | **Get ready to eat healthy!:**   - What is healthy eating? - Why is healthy eating important to you? - How can you use your personal strengths to eat healthier? | **A healthy plate:**   - Wholegrains, proteins & fruits and vegetables - Serving sizes - Setting SMART eating goals | **Let’s go grocery shopping!:**   - How do we make food related decisions? - Reading and understanding food labels | **Be a food detective:**   - What is food tracking? - Different ways we can track our food - Serving size hacks | **Stick to your healthy eating goals:**   - Different social eating situations - Common challenges to healthy social eating - Strategies to stick to your healthy eating goals in social situations | **Slippery slope of maintaining healthy eating:**   - Overcoming diet slips and negative self-judgement - Eat Well recap and wrap-up |
| Stress Less | **Get ready to manage your stress!**:   - What is stress? - Why is managing stress important to you? - How can you use your personal strengths to better manage stress? | **Meet your emotions:**   - Why do we experience emotions? - The 4 core emotions - Complex emotional combinations | **It’s more than just feelings:**   - The thoughts-feelings-behaviours cycle - The function of our thoughts - Emotional feelings and physical sensations - What we do and don’t do | **The emotional journey:**   - What comes before an emotional experience? - What happens during an emotional experience? - What happens after an emotional experience? | **Life’s a Breeze:**   - What is slow-paced breathing? - The benefits of slow-paced breathing - How to practice slow-paced breathing - Using Breeze as a tool | **Stress busting routine:**   - Stress Less recap and wrap-up - Evening routine for a stress-free sleep |

Table. Structure of a coaching session (example).

| **Dialogue Segment** | **Dialogue Category** | **Example** |
| --- | --- | --- |
| **Start** | Hello Microdialogue | C: Hey there $participantName 👋 // Time for today’s coaching session! |
| **Core Content** | Check on previous behavioural intention (from Sessions 2-6) | C: Just picking up where we left of last time// You chose the following option://  BehaviourIntentionSLDay1//Did you manage to do it $participantName?  U: Yup, all done! 😊: 1 Haven't gotten round to it yet: 2 |
|  | Coaching Session Content | **Meet your emotions:**   - Why do we experience emotions? - The 4 core emotions - Complex emotional combinations |
|  | Set Behavioural Intention | C: Which of these would you like to do?   1. I will reflect on the functions my emotions have served 2. I will review the four core emotions 3. I will write a journal entry about an emotional incident |
| **End** | Appointment Setting Microdialogue | C: Around the same time for our next session together? 😊  U: Sounds good: 1 Can I pick another time?: 2 |
|  | Goodbye Microdialogue | C: Take care $participantName!  U: You too $CoachName!: 1 Thanks 🙂: 2 |

Table. Overview of all life hacks in LvL UP.

| **Move More** | **Eat Well** | **Stress Less** |
| --- | --- | --- |
| **1** The Sunshine Vitamin | **17** Eat The Rainbow | **33** What’s Your Favourite Song? |
| **2** Break It Up | **18** Sui Dai, Please! | **34** Get Outside and into Nature |
| **3** TV Exercise | **19** The Power of Wholegrains | **35** Pay It Forward |
| **4** Ditch the Car | **20** Take Small Bites | **36** Self-Affirmation |
| **5** Every Minute Counts | **21** Thought For Food | **37** Make Time for Loved Ones |
| **6** Explore Your Surroundings | **22** Try Healthier Cooking Methods | **38** Wish Someone A Good Day |
| **7** Schedule Your Exercise | **23** Mindful Eating | **39** Write it Down |
| **8** Two Birds, One Stone | **24** Cut Down on Salt | **40** Reading Break |
| **9** Exercise Buddy | **25** Avoiding Processed Foods | **41** Master your Notifications |
| **10** Feel-Good Exercise | **26** Healthy Snacks on The Go | **42** Happy Memories |
| **11** Lunchtime Activity | **27** Stay Hydrated | **43** Have A Laugh |
| **12** Stretch to a Clearer Mind | **28** More Veggies Please! | **44** Practice Gratitude |
| **13** Don’t Just Wait Around | **29** Beware of Hidden Calories | **45** Out of Sight, Out of Mind |
| **14** Elevate Yourself | **30** Dabao | **46** Say “No” To Caffeine |
| **15** Exercise Motivation | **31** Chew with Joy | **47** Set Your Intention |
| **16** Walk In Place | **32** Suss Out Saturated Fat | **48** Declutter Your Space |

Table. Description of the LvL UP’s overarching story, four episodes, and example of the different mini-stories that are presented for each character.

| Overarching story: *‘A company has a huge upcoming product launch. Kai, Dana and Ray have to work overtime a lot, which leads to tension between Kai and his girlfriend Alex, tensions between Dana and her husband and kids, and affects Ray’s physical and mental health.’* |
| --- |
| **Season 1 Episode 1: Stressed and Overworked**  Duration: 3min 22sec  Synopsis: With the product launch getting closer, Kai doesn’t show up for his date with Alex, Dana misses out on family time, and Ray survives off coffee and instant noodles. |
| **Season 1 Episode 2: Something’s Got to Change**  Duration: 2min 09sec  Synopsis: It’s the weekend and Kai’s date with Alex gets interrupted by a work call, Ray neglects eating, sleeping and socializing, and Dana’s son is upset that she can’t spend time with him. |
| **Season 1 Episode 3: Taking Steps**  Duration: 3min 08sec  Synopsis: Alex decides she needs time and space to figure out things for herself, Ray helps Kai through a tough time, and the boys stand up for Dana when she needs it. |
| **Season 1 Episode 4: Reaching New Levels**  Duration: 4min 12sec  Synopsis: It’s launch day! Kai, Alex, Dana, and Ray meet at the launch party and reflect on their ongoing journey to LvL**^UP^** their body and mind, and become the best versions of themselves. |


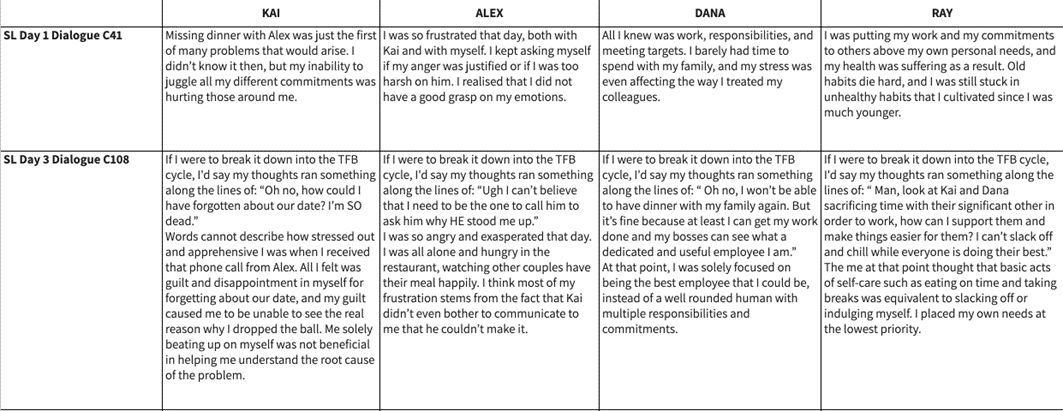


Table. Overview of the LvL UP’s shield logic.

| **Level** | **Task Type** | **Tasks needed for progression** | **Puzzle Piece award criteria** | **Award** | **Progress to** |
| --- | --- | --- | --- | --- | --- |
| Level 1 | LvL UP Coaching Sessions | Complete any 6 coaching sessions with Chatbot | Completion of unique pillar-specific coaching session | 1 puzzle piece/coaching session (yellow) | Level 2 upon collecting all 14 puzzle pieces (animation of new shield + LvL UP Episode 2 unlocked) |
|  | LvL UP Basics | Download LvL UP App | Download LvL UP from App Store | 1 puzzle piece (light green) |  |
|  |  | Complete “Welcome Dialogue” | Complete “Welcome Dialogue” which includes pre-test “Vulnerability Assessment” | 1 puzzle piece (light green) |  |
|  |  | Complete “About Me (Part 1)” survey via Limesurvey | Complete demographic questionnaire on Limesurvey | 1 puzzle piece (light green) |  |
|  |  | Complete “LvL UP Booklet” interest check survey | Complete “LvL UP Booklet interest check” on Limesurvey | 1 puzzle piece (light green) |  |
|  |  | Complete “Technology Acceptance” questionnaire | Complete “Technology Acceptance” questionnaire on Limesurvey (only unlocked after 1 coaching session has been completed) | 1 puzzle piece (light green) |  |
|  |  | *Complete “Check-In 1” Session | Complete “LvL 1 Review” Chatbot dialogue | 1 puzzle piece (light green) |  |
|  | LvL UP Life Hacks | Implement at least 1 life hack | Complete any one life hack activity that has not been done by user previously | 1 puzzle piece (dark green) |  |
|  | LvL UP Tools | Use Breeze or Journaling at least once | To complete 1 Breeze training session OR save 1 Journal entry | 1 puzzle piece (purple) |  |
| Level 2 | LvL UP Coaching Sessions | Complete any 6 coaching sessions with Chatbot | Completion of unique pillar-specific coaching session | 1 puzzle piece/coaching session (yellow) | Level 3 upon collecting all 14 puzzle pieces (animation of new shield + LvL UP Episode 3 unlocked) |
|  | LvL UP Basics | Complete “About Me (Part 2)” survey via Limesurvey | Complete SES questionnaire on Limesurvey | 1 puzzle piece (light green) |  |
|  |  | Complete “Working Alliance” questionnaire | Complete “Working Alliance” questionnaire on Limesurvey | 1 puzzle piece (light green) |  |
|  |  | Complete “Willingness to Pay” questionnaire | Complete “Willingness to Pay” questionnaire on Limesurvey | 1 puzzle piece (light green) |  |
|  |  | *Complete “Check-In 2” Session | Complete “LvL 2 Review” Chatbot dialogue | 1 puzzle piece (light green) |  |
|  | LvL UP Life Hacks | Implement a life hack | Complete any one life hack activity that has not been done by user previously | 1 puzzle piece (dark green) |  |
|  |  | Implement a life hack | Complete any one life hack activity that has not been done by user previously | 1 puzzle piece (dark green) |  |
|  | LvL UP Tools | Use Breeze or Journaling | Complete 1 Breeze training session OR save 1 journal entry | 1 puzzle piece (purple) |  |
|  |  | Use Breeze or Journaling | Complete 1 Breeze training session OR save 1 journal entry | 1 puzzle piece (purple) |  |
| Level 3 | LvL UP Coaching Sessions | Complete any 6 coaching sessions with Chatbot | Completion of unique pillar-specific coaching session | 1 puzzle piece/coaching session (yellow) | Final level completed upon collecting all 14 puzzle pieces (final celebratory animation + LvL UP Episode 4 unlocked) |
|  | LvL UP Basics | Complete “Cultural Adaptation” questionnaire | Complete “Cultural Adaptation” questionnaire via Limesurvey | 1 puzzle piece (light green) |  |
|  |  | *Complete “Thank You Dialogue” | Complete “Thank You Dialogue” which includes post-test “Vulnerability Assessment” | 1 puzzle piece (light green) |  |
|  | LvL UP Life Hacks | Implement a life hack | Complete any one life hack activity that has not been done by user previously | 1 puzzle piece (dark green) |  |
|  |  | Implement a life hack | Complete any one life hack activity that has not been done by user previously | 1 puzzle piece (dark green) |  |
|  |  | Implement a life hack | Complete any one life hack activity that has not been done by user previously | 1 puzzle piece (dark green) |  |
|  | LvL UP Tools | Use Breeze or Journaling | Complete 1 Breeze training session or save 1 journal entry | 1 puzzle piece (dark green) |  |
|  |  | Use Breeze or Journaling | Complete 1 Breeze training session OR save 1 journal entry | 1 puzzle piece (dark green) |  |
|  |  | Use Breeze or Journaling | Complete 1 Breeze training session OR save 1 journal entry | 1 puzzle piece (dark green) |  |

Figure. Overview of the different routes and combinations of notifications users may receive in relation to coaching sessions.


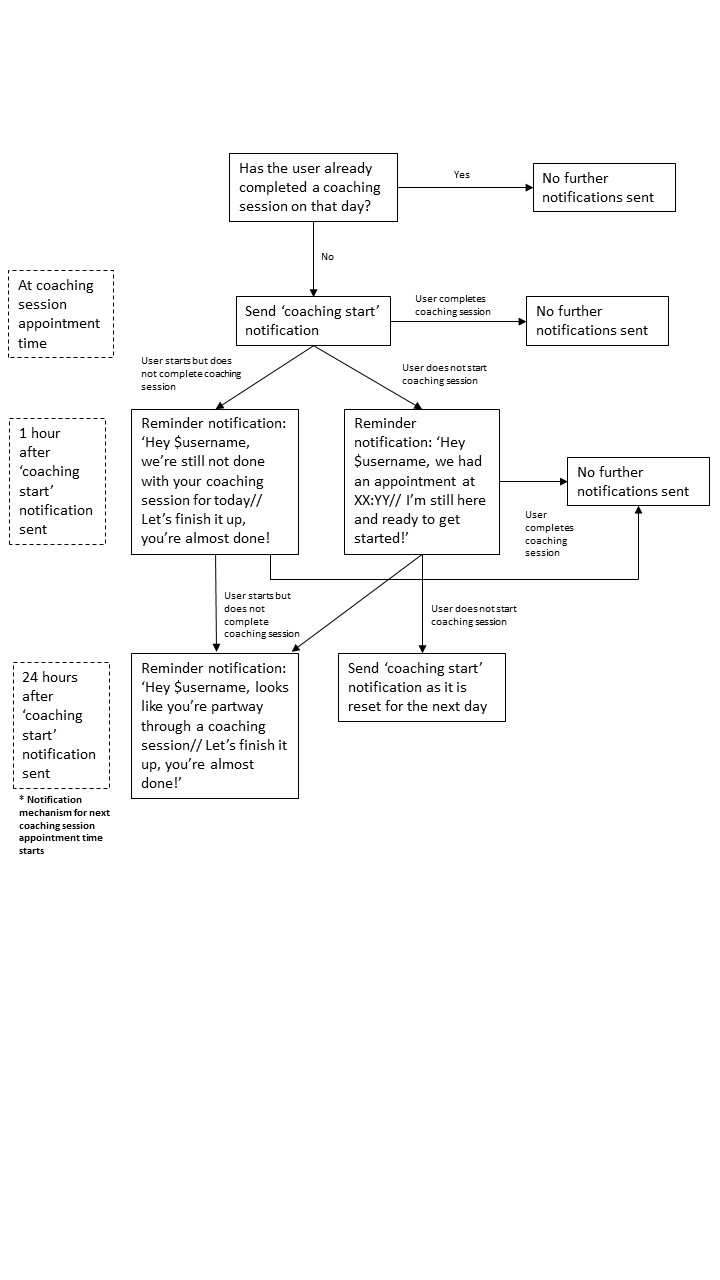


Figure. Overview of the different routes and combinations of notifications users may receive in relation to Life Hacks.


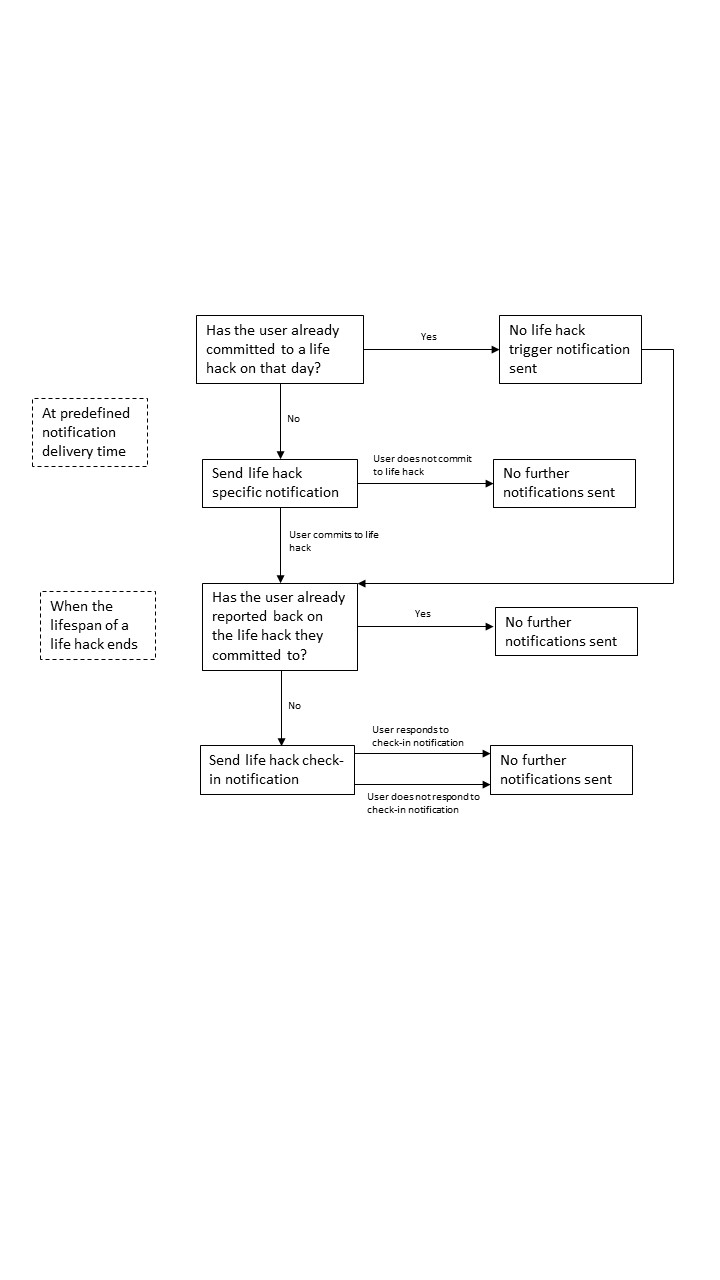


Table. Overview of the different dropout and disengagement notifications in LvL UP.

| **Hours after ‘Coaching Start’ notification sent** | **Action** | **‘Not started Coaching Session’ content** | **‘Started but did not complete Coaching Session’ content** |
| --- | --- | --- | --- |
| 1 hour | Send coaching reminder notification 1 | Coach: Hi $participantName, we had a coaching appointment at 7.30pm. I’m still here and ready to get started! $coachName | Coach: Hi $participantName, we’re still not done with our coaching session for today. Let’s finish it up, you’re almost done! $coachName |
| 24 hours | Send coaching reminder notification 2 | Coach: Hi $participantName, it’s 7.30pm and time for our coaching session. Ready to start when you are! $coachName | Coach: Hi $participantName, looks like you’re partway through a coaching session. Let’s finish it up, you’re almost done! $coachName |
| 72 hours | Send coaching reminder notification 3 | Coach: Hi $participantName, time for our scheduled coaching appointment at 7.30pm. Let’s get started! $coachName | Coach: Hi $participantName, I see that you’re partway through a coaching session. Let’s finish it up, not long left! $coachName |
| Day 5 | Send coaching reminder email 1 | Coaching Reminder Email 1 (see section 7.2.3) | Coaching Reminder Email 1 (see section 7.2.3) |
| Day 7 | Send coaching reminder email 2 | Coaching Reminder Email 2  (see section 7.2.3) | Coaching Reminder Email 2  (see section 7.2.3) |

| ***Days of no app activity** | **Actions** | **Content** |
| --- | --- | --- |
| **Day 7 | send Jump back in Email | Jump back in Email (see section 7.2.3) |
| Day 14 | send Jump back in Email | Jump back in Email (see section 7.2.3) |
| Day 30 | send Final Dropout Email | ***Final Dropout Email (see section 7.2.3) |
