# Supplementary material for: Development of “LvL UP 1.0”: a smartphone-based, conversational agent-delivered holistic lifestyle intervention for the prevention of non-communicable diseases and common mental disorders
Source: Front Digit Health. 2023 May 10;5:1039171. doi: 10.3389/fdgth.2023.1039171 (PMC10207359; doi:10.3389/fdgth.2023.1039171)

Figure. A selection of screenshots from LvL UP app, including (in order) coaching session excerpts, shield, example life hack, breeze, and journal.


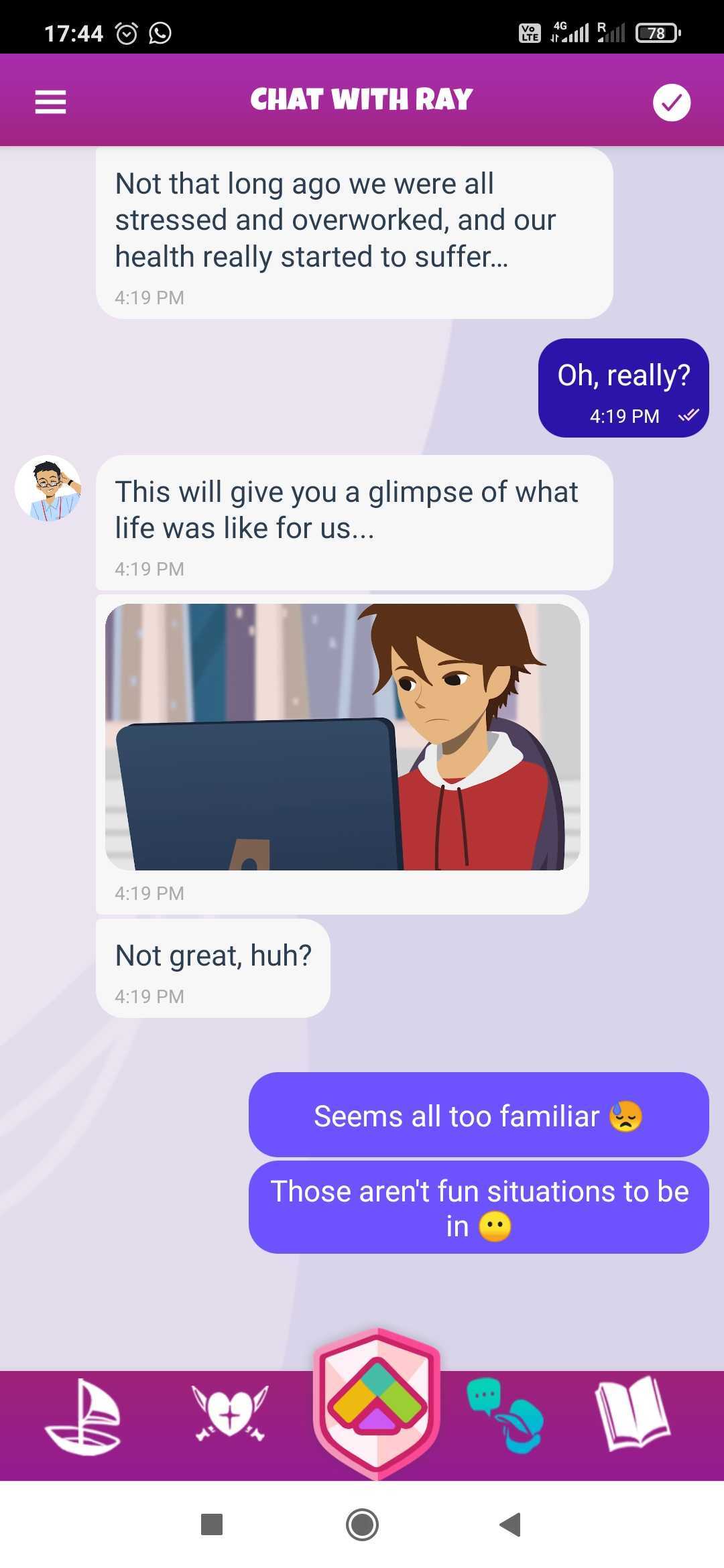

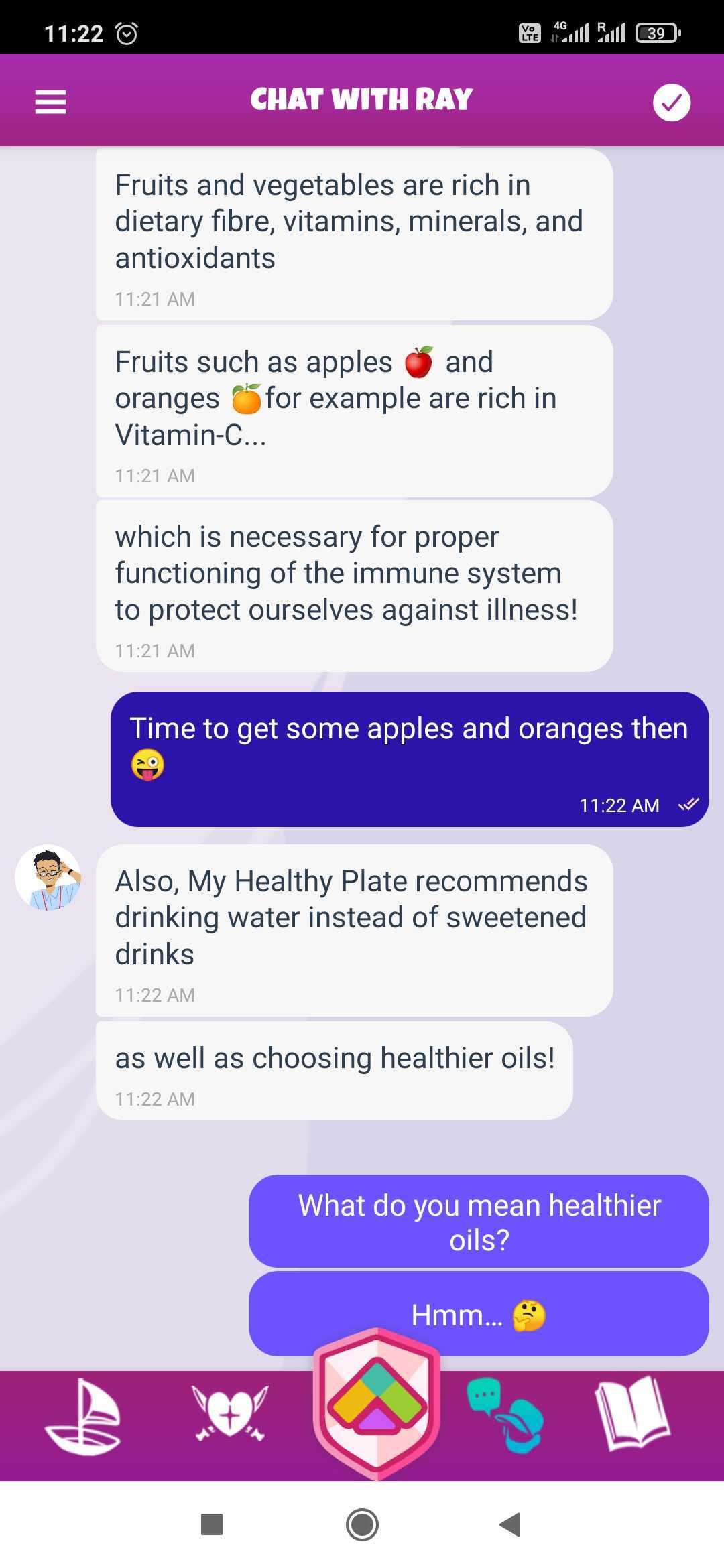


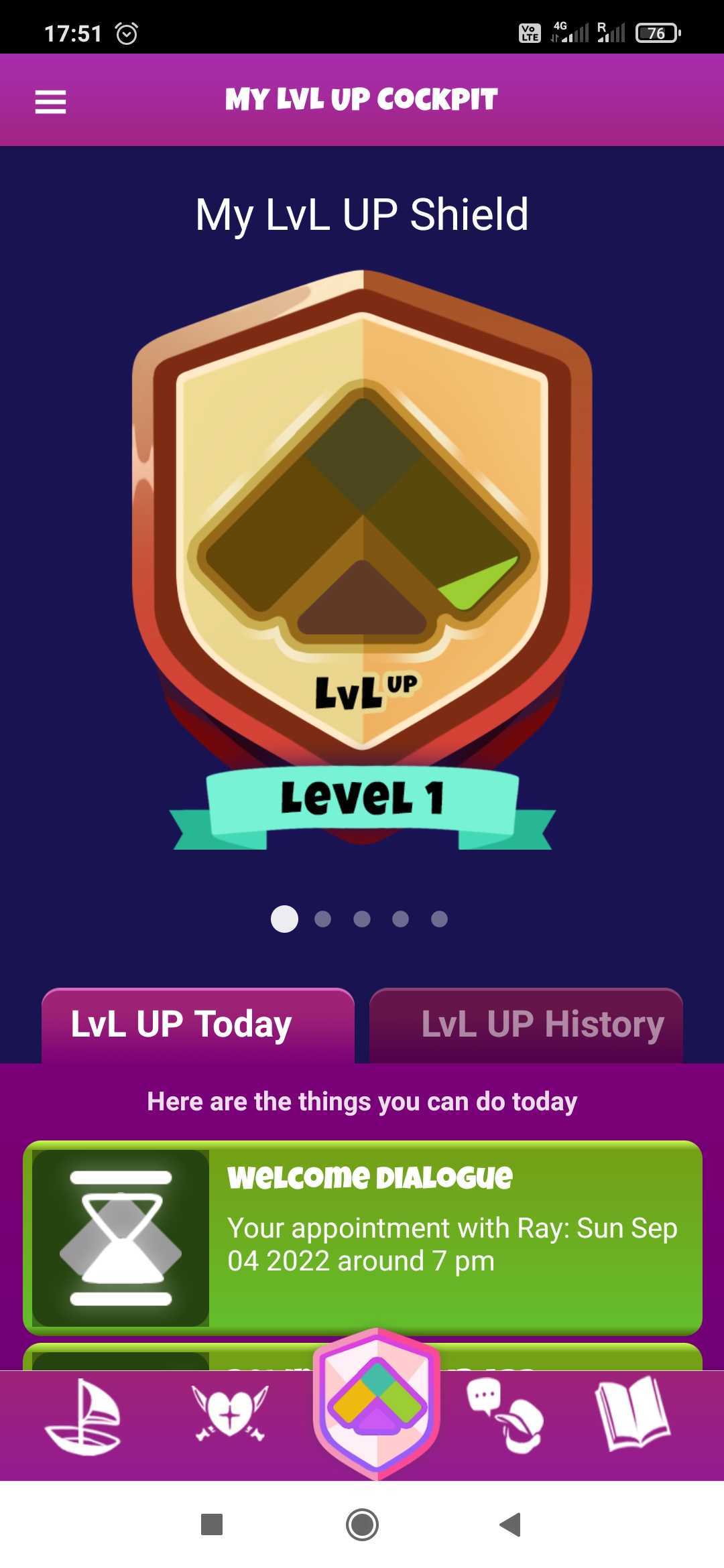

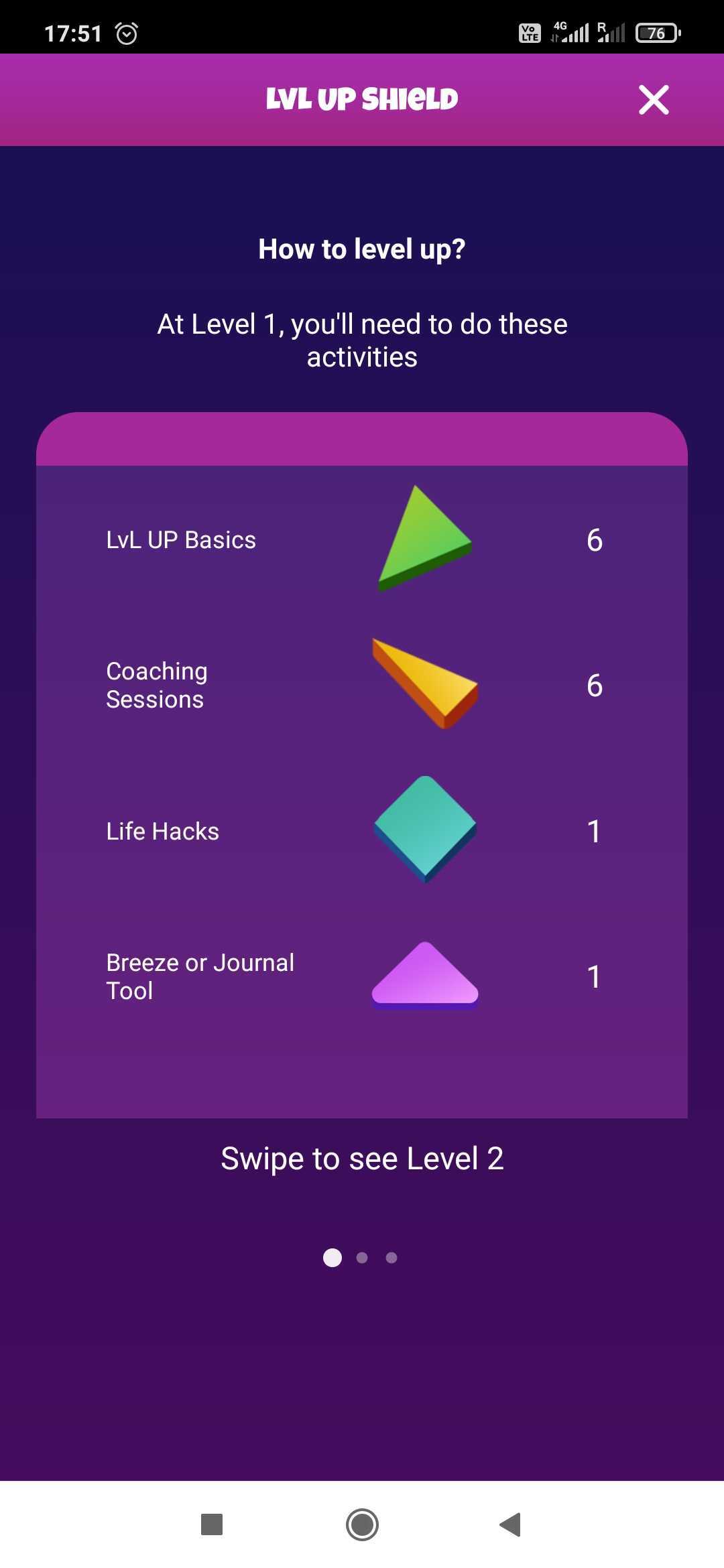


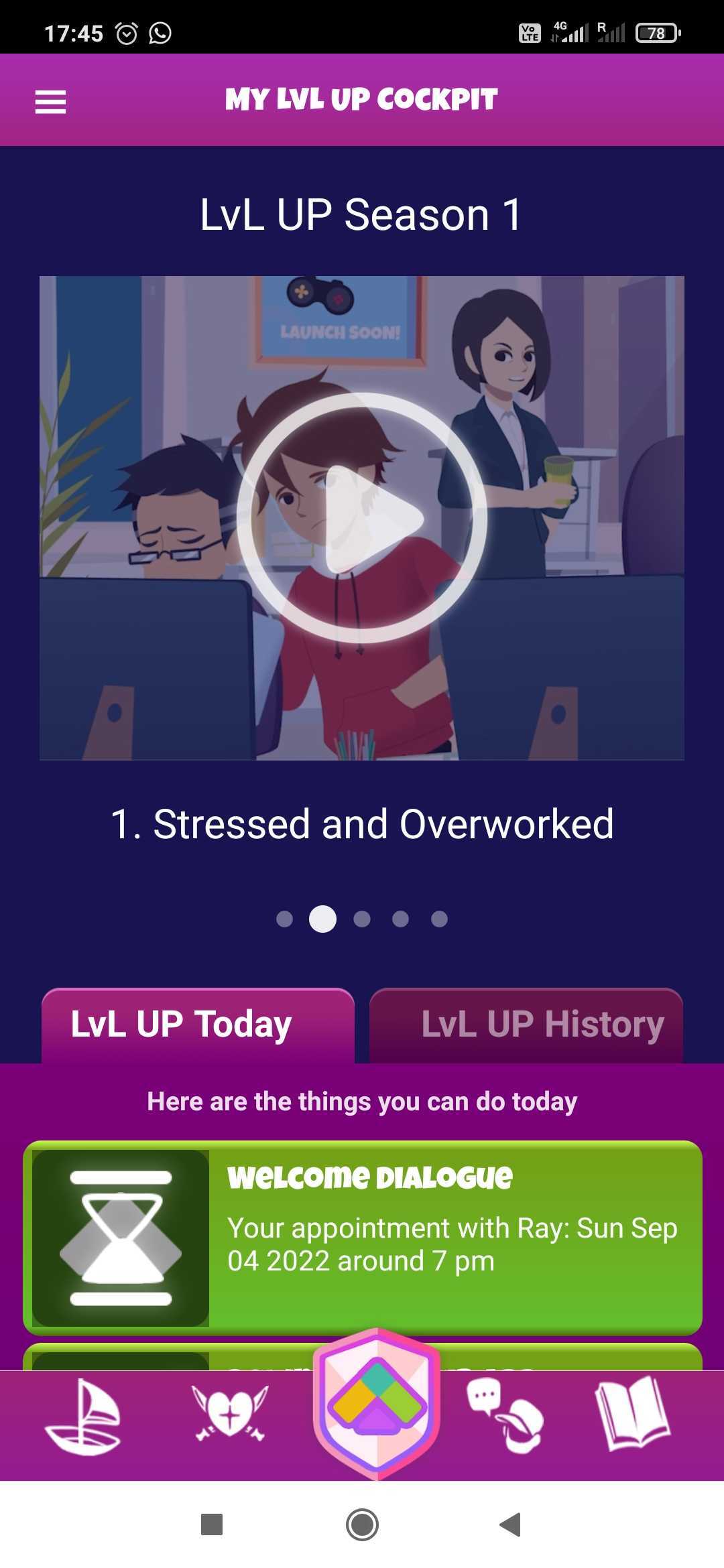

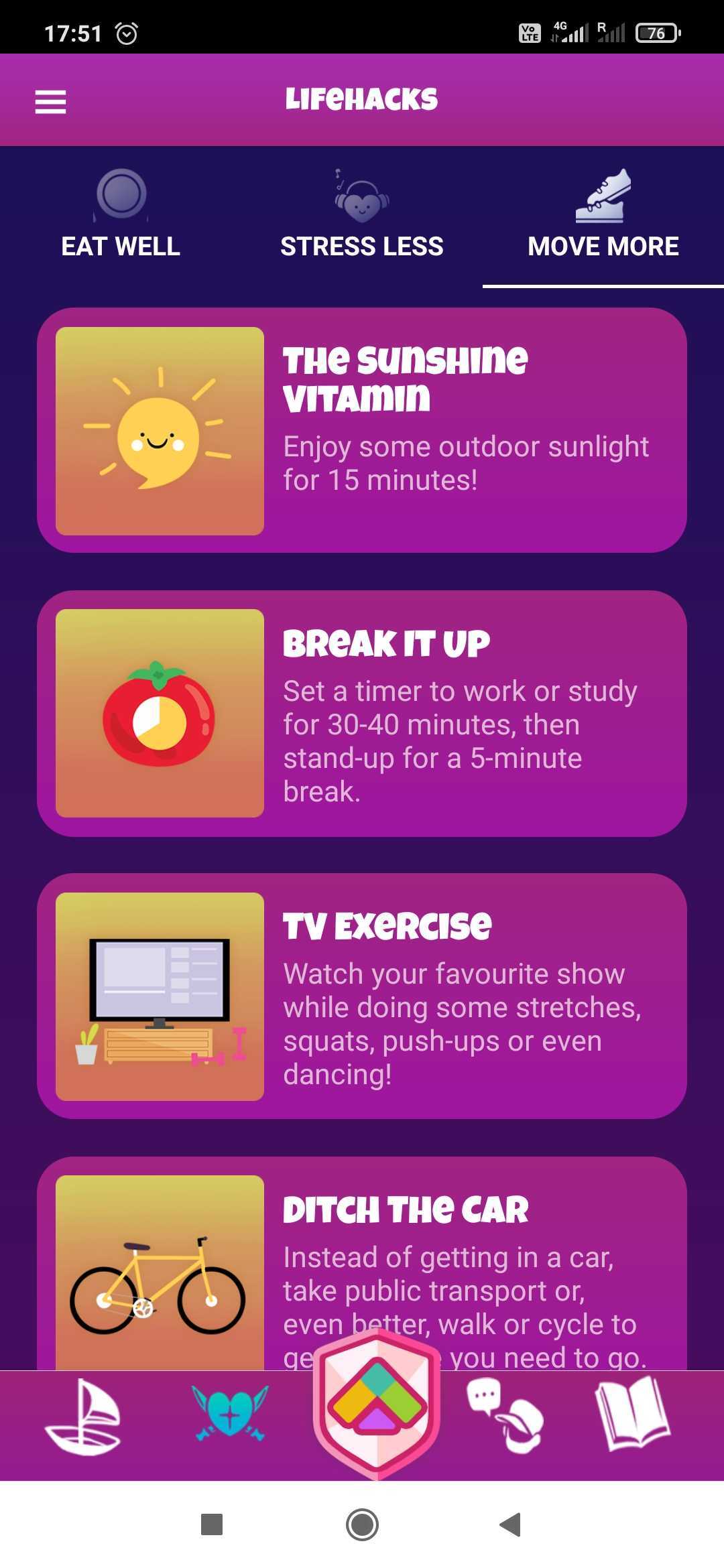


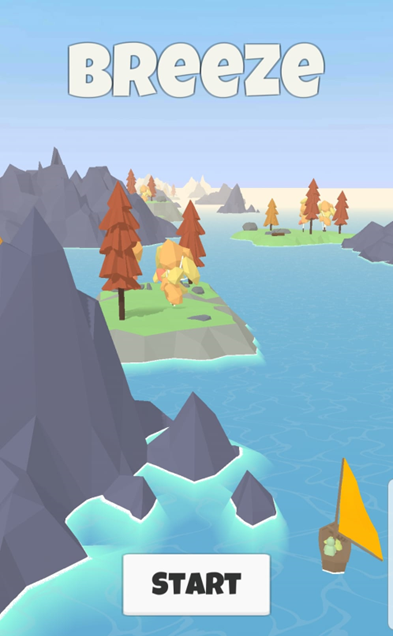

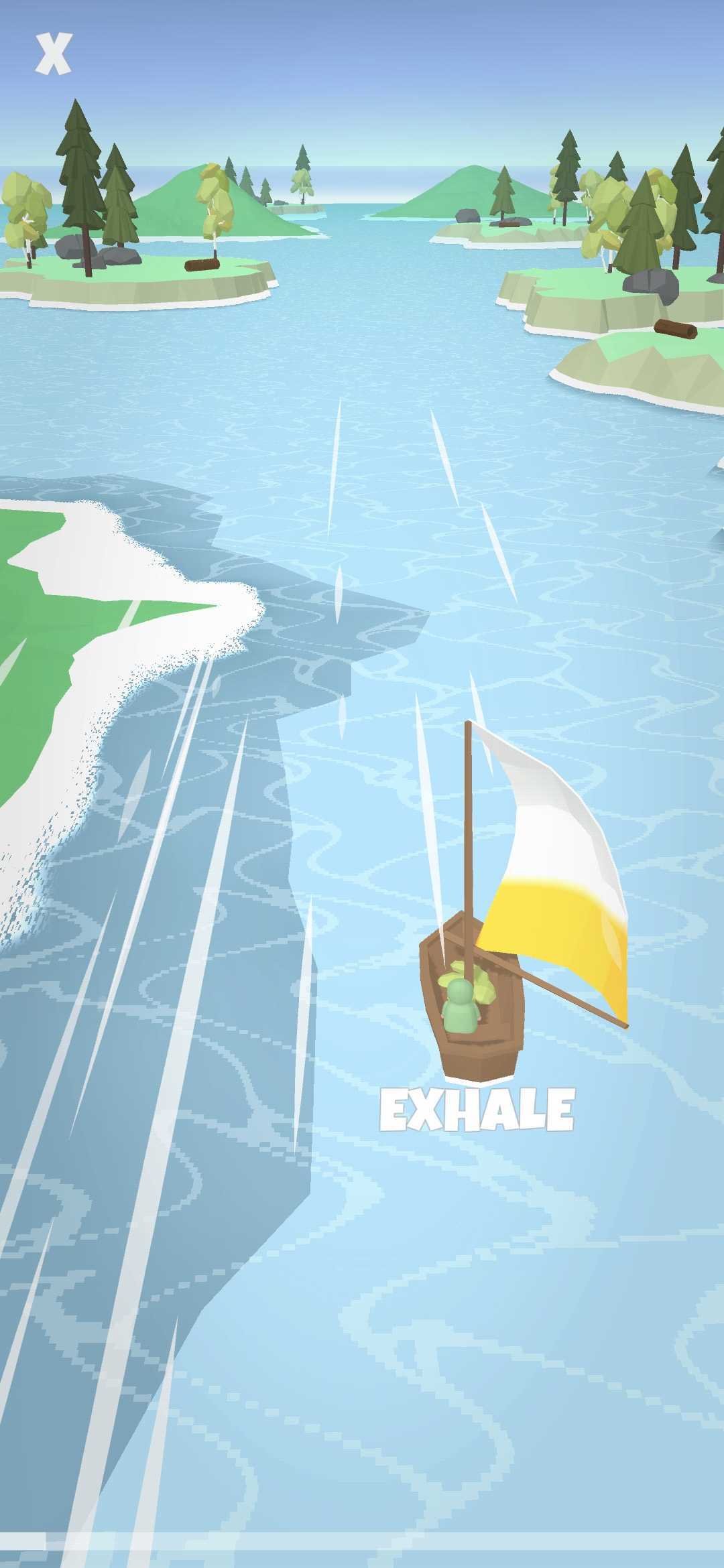


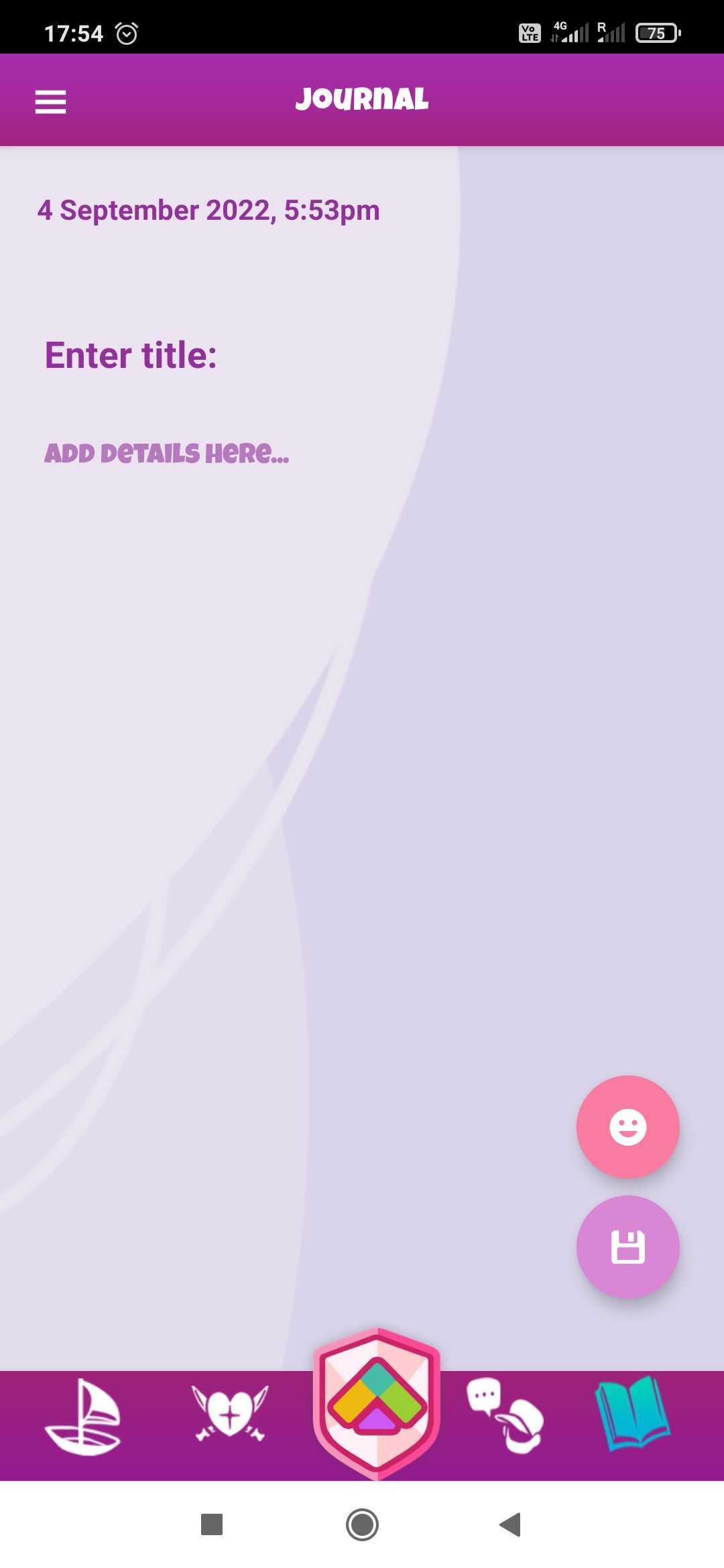

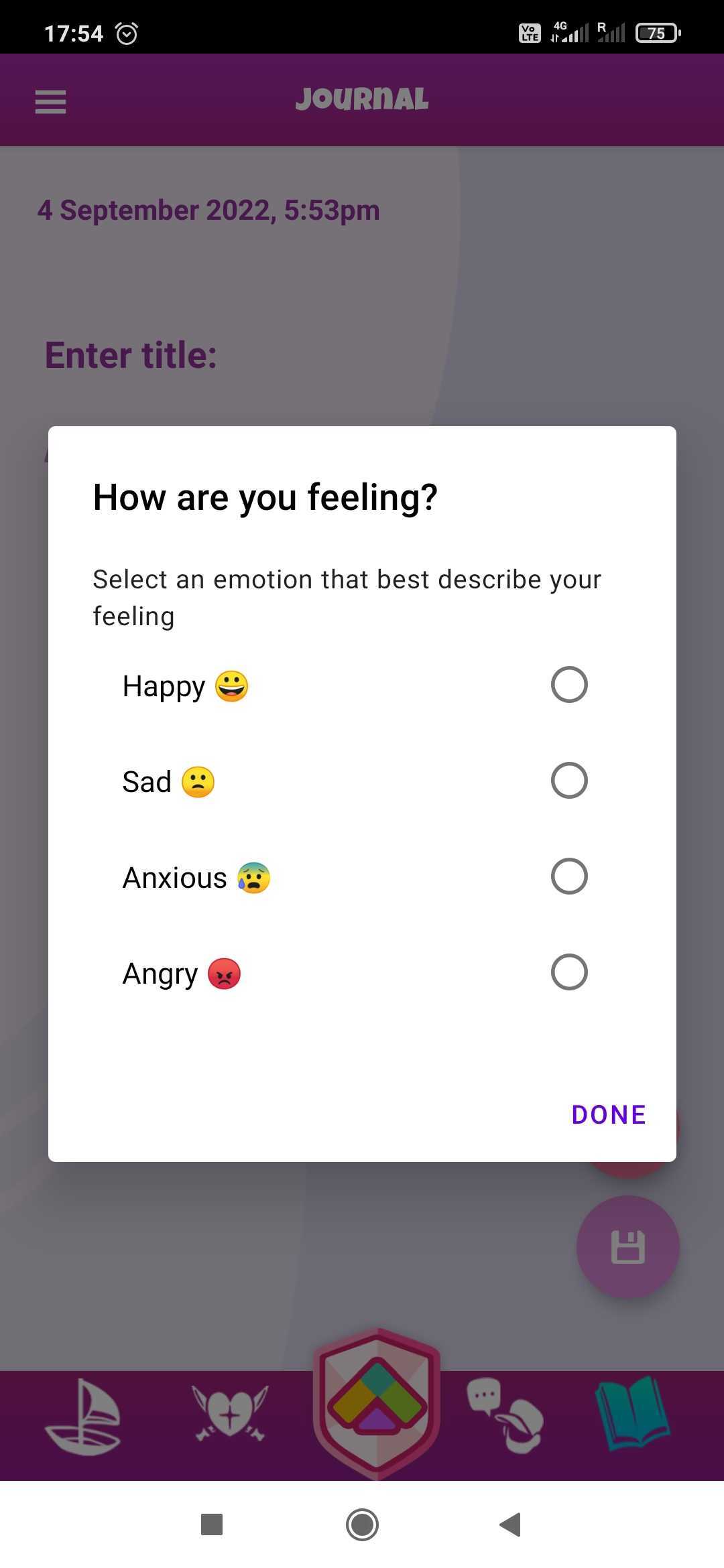

Supplement: Supplementary file 1 [file Datasheet1.zip › Supplementary File 3.DOCX]
