# Supplementary material for: Development of “LvL UP 1.0”: a smartphone-based, conversational agent-delivered holistic lifestyle intervention for the prevention of non-communicable diseases and common mental disorders
Source: Front Digit Health. 2023 May 10;5:1039171. doi: 10.3389/fdgth.2023.1039171 (PMC10207359; doi:10.3389/fdgth.2023.1039171)
Supplement: Supplementary file 1 [file Datasheet1.zip › Supplementary File 4.PDF]

LvL UP

Ready to LvL UP your life?

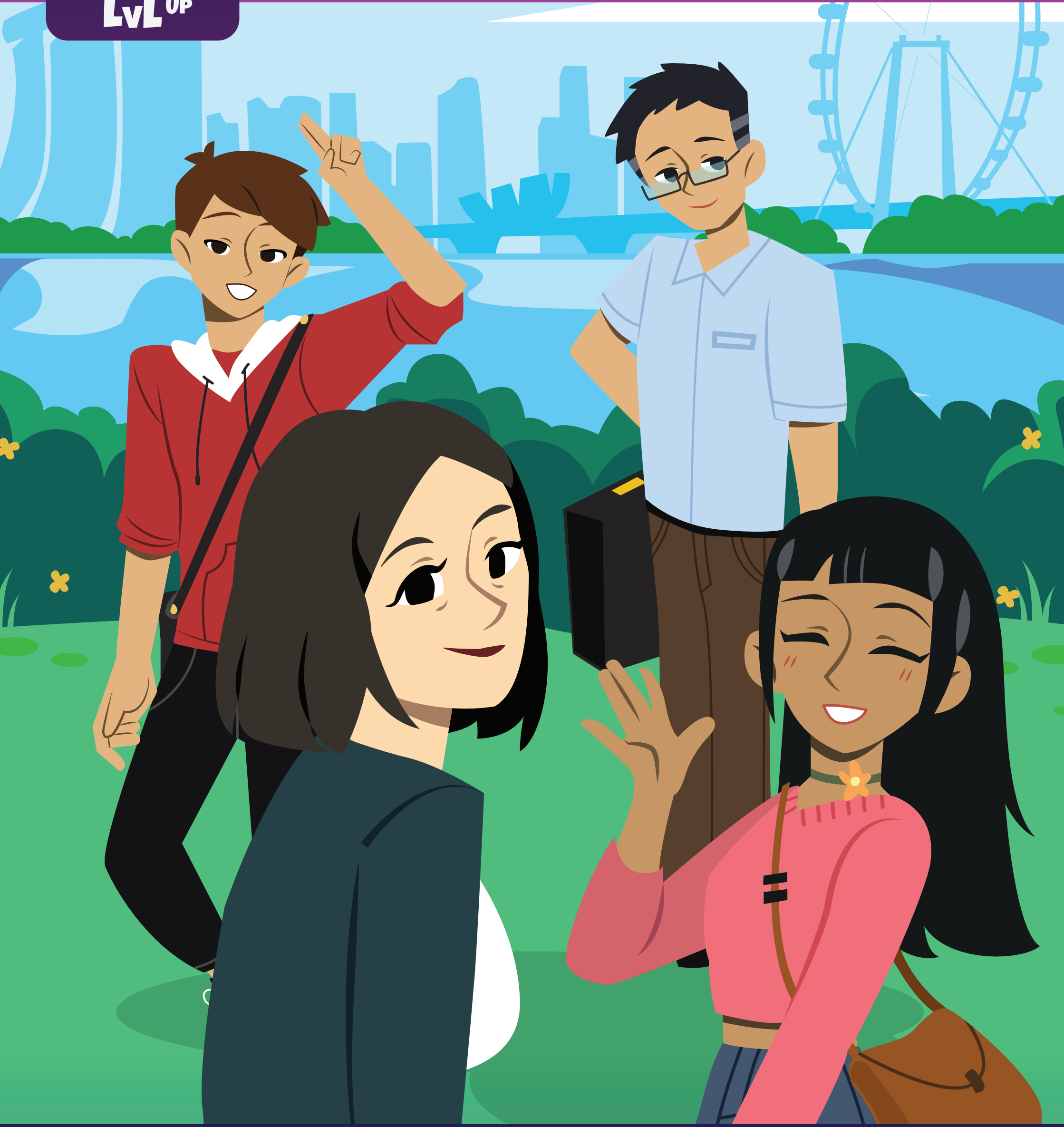

Join Kai, Alex, Dana and Ray on a journey to strengthen your body and mind

9 in 10

Singaporeans are struggling with their health and wellbeing

Be the other 1

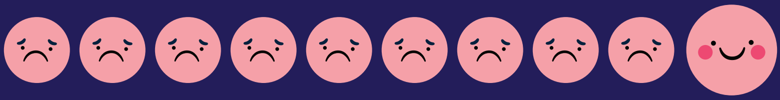

NUS National University of Singapore

LEE KONG CHIAN SCHOOL OF MEDICINE

(SEC) SINGAPORE-ETH CENTRE

ETH zürich

LIONFISH STUDIOS

Welcome to LvLUP

Hi there!👋

A very warm welcome to the LvLUP booklet! It's so great to have you here and I thank you for taking the time and energy to have a look through. LvLUP is all about strengthening your body and mind to become the best version of yourself. We know that our body and mind work together in so many complex ways, and focusing on one without the other would only address one of the many intricate parts that make us who we are – strong, determined, and unique individuals who want to LvLUP our lives. 🤩

Your LvLUP journey will take you through three pillars; the Move More pillar focusing on physical activity, the Eat Well pillar on nutrition and healthy eating habits, and the Stress Less pillar on stress management and emotional wellbeing. Here's the best part – you can choose how you'd like to move through these pillars, as we know that everyone has their own path to reach their destination. What's more, is that you won't be going on this journey alone. We have Kai, Alex, Dana, and Ray, ordinary people just like you and me, who will share their experiences of their ongoing journey to LvLUP their body and mind.

Even though LvLUP comes as an app that you can download to your smartphone, we value the importance of being offline too, so we have created this LvLUP booklet. There are two sides to every coin, and as technology advances and opens up so many doors to things that weren't previously possible, it can also be a source of stress when we constantly feel the need to be plugged in. This booklet gives you the option to experience some of the tools and features that you can find on the LvLUP app without needing access to your smartphone or any other device. We recommend using both the app and the booklet to get the best of both worlds and the full LvLUP experience, but of course, it is completely up to you to decide!

Over the past 18 months, the LvLUP team has been working hard to create a platform that can fit seamlessly into your busy life. Through a combination of the latest evidence-based research, discussions with potential users and interviews with industry experts, and working together with a local game development studio, we have come up with this first version of LvLUP. We have big plans for the future of LvLUP and are so excited to start working on those too! But for that, we need your help. LvLUP can only be as good as you, our users, find it to be. Your comments and feedback are what will drive the changes and developments made to the next version of LvLUP and beyond!

Whether you choose to download the LvLUP app, use this booklet, or do both, I hope you are able to benefit from your experience in whatever way that may be, and most importantly, enjoy the journey! I look forward to hearing about it . 😉

Download on the App Store

GET IT ON Google Play

Scan QR code to download the app

Have fun exploring!
On behalf of the LvLUP team,

Aishah Alattas

LvLUP is part of the research of the Future Technologies Programme, Singapore-ETH Centre. Any data given will be used solely for future research purposes and will be kept strictly confidential.

TABLE OF CONTENTS

- MEET KAI, ALEX, DANA AND RAY
- LIFE HACKS
- SLOW-PACED BREATHING
- JOURNAL
- LvL^UP APP
- MEET THE LvL^UP TEAM

Meet the coaches

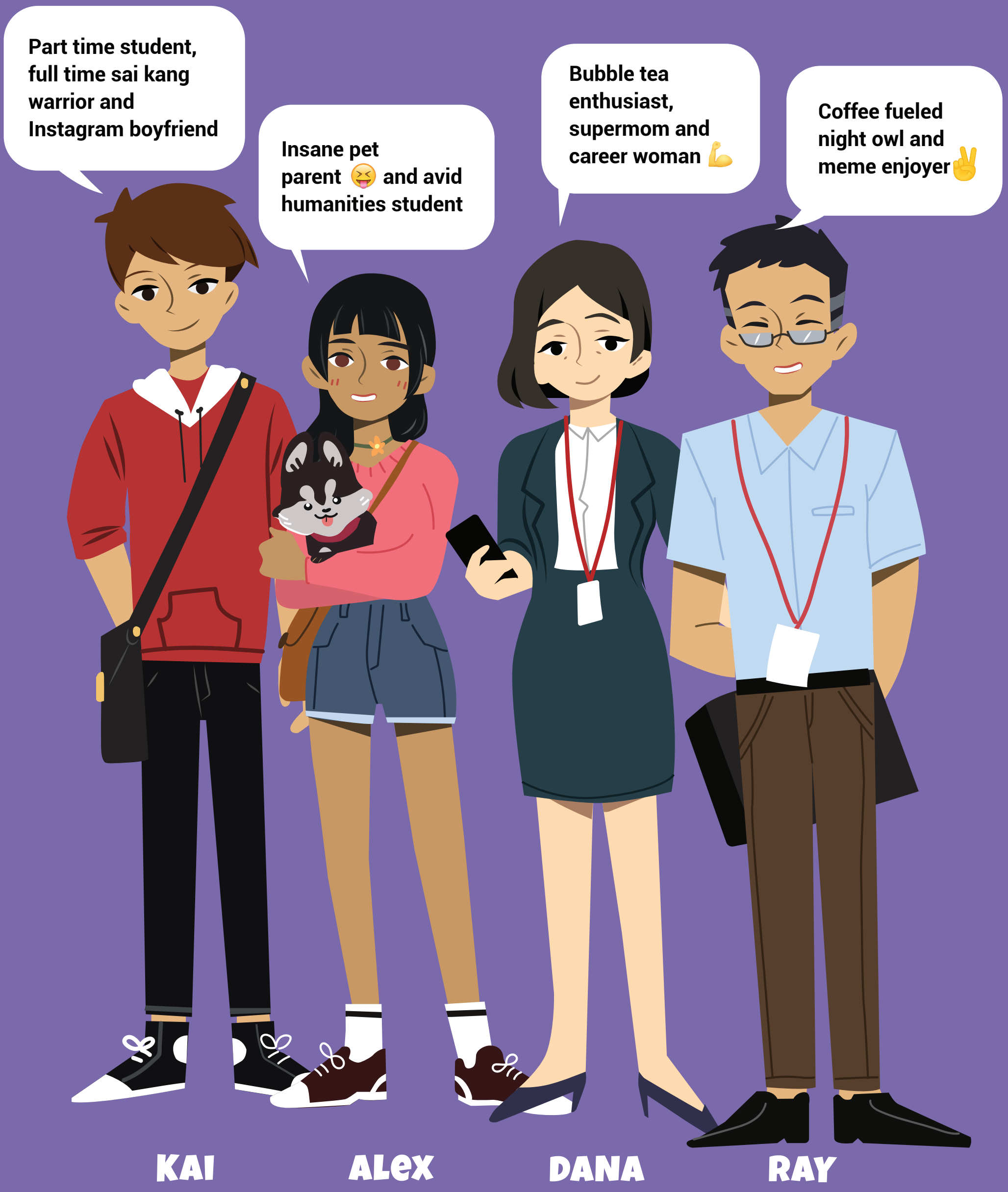

LIFE HACKS

Strengthening our body and mind doesn't always mean drastic changes that take a lot of time, energy and effort.

The small steps we take daily can build up to produce great effects! Life Hacks have been particularly helpful for me with juggling commitments and myself.
The activities are quick and easy, and it feels so good when I tick something off my to-do list!

On these next pages, you will find a compilation of the life hacks that come under our three pillars.

You can cut out the individual cards to act as convenient reminders. Even if a life hack comes under one of these pillars, many of them produce benefits for other pillars too!

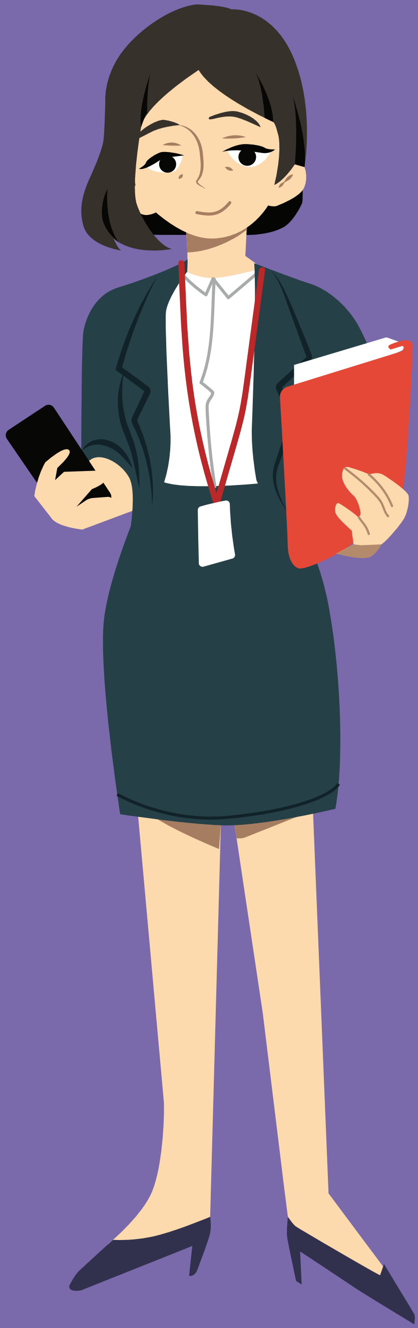

LIFE HACKS: EAT WELL

EAT THE RAINBOW

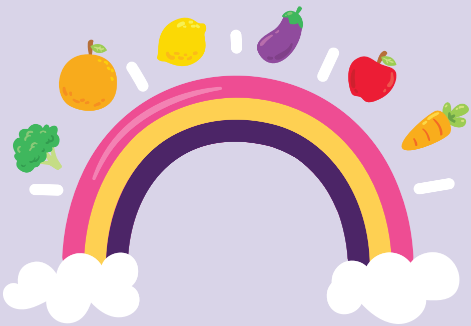

SIU DAI PLEASE!

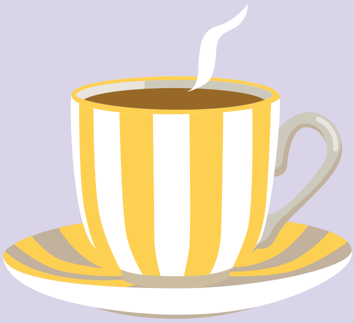

THE POWER OF WHOLEGRAINS

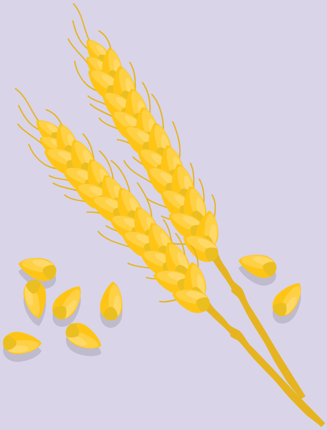

TAKE SMALL BITES

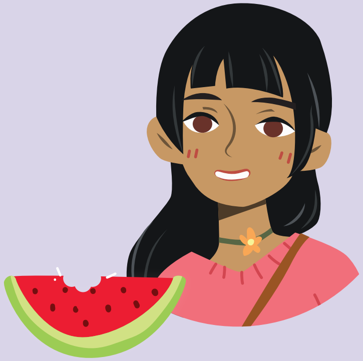

Ask for 'Siu Dai' at the coffee shop to lower the sugar content in your drink by about 1-1.5 teaspoons, or do this yourself if making your drink at home.

Red, orange, green, purple, yellow, and white!

Eat at least two different colour of fruits and veggies in your meals!

Take small bites of your food instead of making your mouth "full".

This allows you to savour the taste of your food and stay fuller for longer!

Add healthy wholegrains like oats, corn, barley, quinoa, brown rice, chappati, wholemeal pasta, bulgar, and millet to your meals.

LIFE HACKS: EAT WELL

THOUGHT FOR FOOD

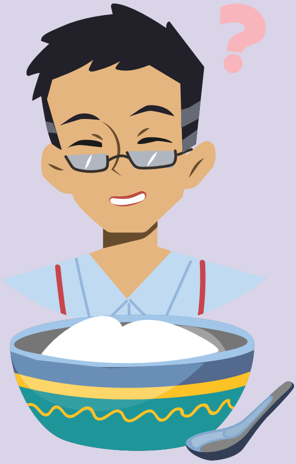

TRY HEALTHIER COOKING METHODS

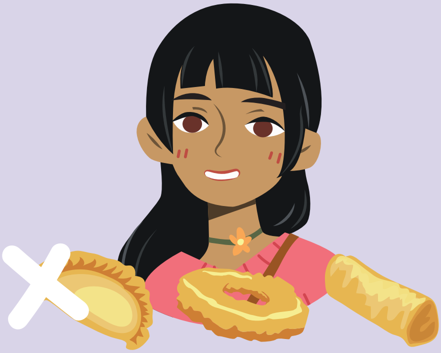

MINDFUL EATING

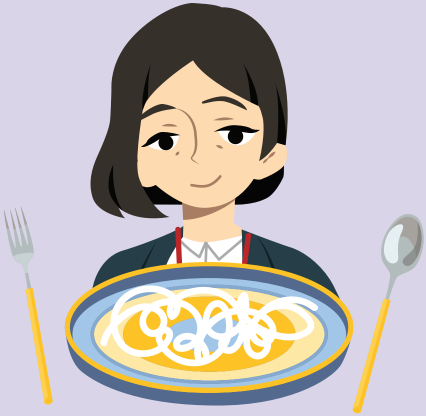

CUT DOWN ON SALT

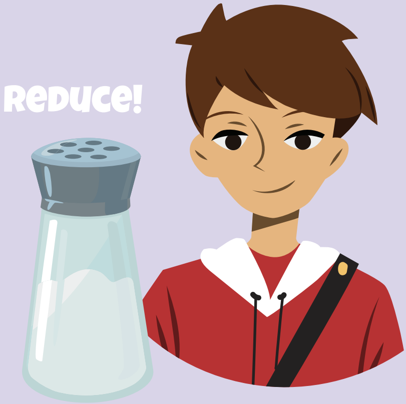

Choose alternative cooking styles such as steamed, baked, braised, roasted, or grilled instead of deep fried dishes.

Pause and ask yourself why you reach out for this food.

What are you feeling right now? Are you physically hungry that you want this food badly?

Season your food naturally with herbs and spices, or go for low salt options when eating out or grocery shopping.

You can easily forget how much you've eaten if you've been mindlessly watching TV or scrolling through your phone.

When you are eating, let it be the only activity you do.

LIFE HACKS: EAT WELL

AVOIDING PROCESSED FOODS

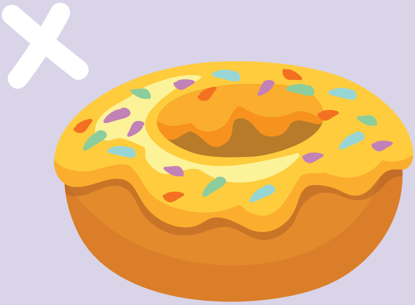

HEALTHY SNACKS ON THE GO

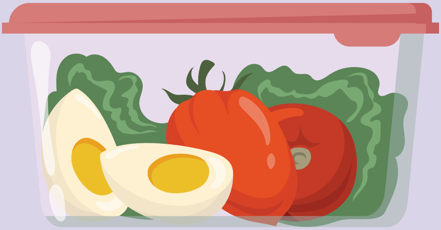

STAY HYDRATED!

MORE VEGGIES PLEASE!

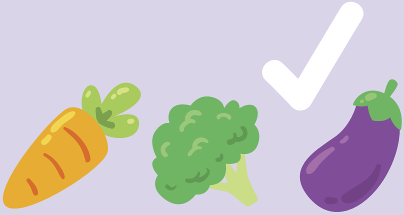

Prepare some healthy snacks before you head out today like pre-cut fruits or veggies, greek yoghurt, or boiled eggs.

Avoid highly processed foods like white rice, white bread, instant noodles, bubble tea, cakes, and candies.

Veggies are rich in vitamins, antioxidants, nutrients and fibre.

Increase your intake of veggies and have at least 2 servings per day where they fill half your plate.

Carry a water bottle to help you stay hydrated.

P.S. if you feel thirsty, you're already dehydrated!

LIFE HACKS: EAT WELL

Beware of hidden calories

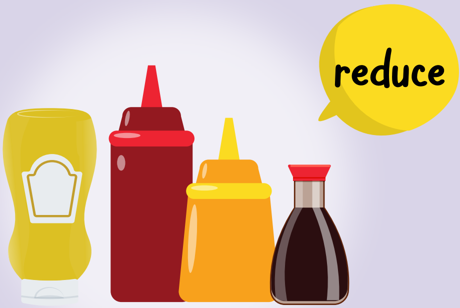

DABAO!

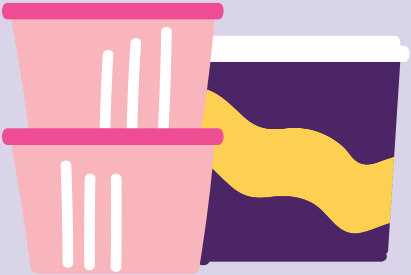

Chew with joy

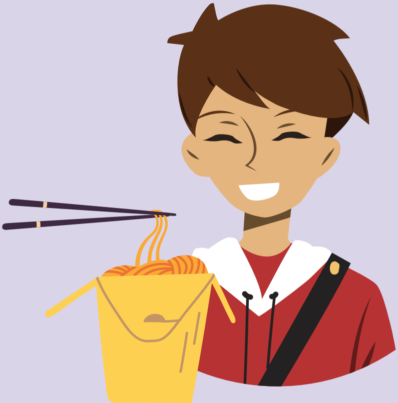

Suss out saturated fat

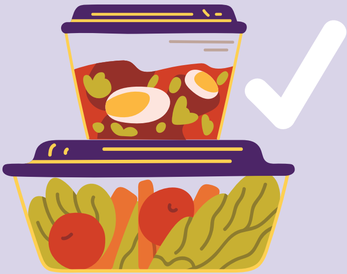

If you're eating out and the portion sizes are too large, ask to dabao the leftovers for the next meal instead of finishing up.

Sauces, gravy, and curry are generally high in calories, salt and sugar. When eating, leave the sauces, gravy, and curry to the side and add only what is needed.

Ask for, or use, less butter, oil, and gravy and remove visible fat and skin from meat to reduce the saturated fat in your meal.

When you eat, smell the aroma of your food and chew each mouthful at least 10 times to boost feelings of fullness and satisfaction.

LIFE HACKS: STRESS LESS

WHAT'S YOUR FAVOURITE SONG?

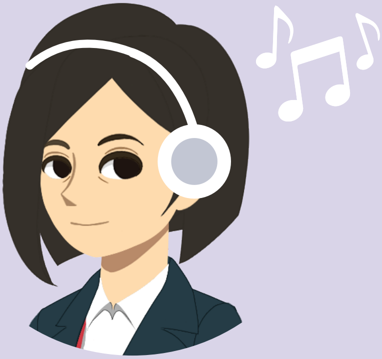

GET OUTSIDE AND INTO NATURE

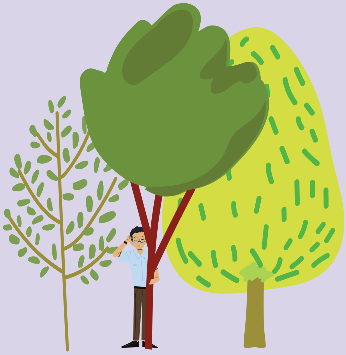

PAY IT FORWARD

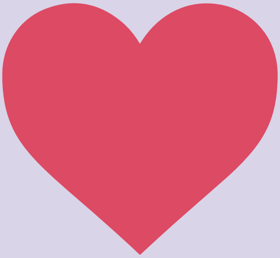

SELF-AFFIRMATION

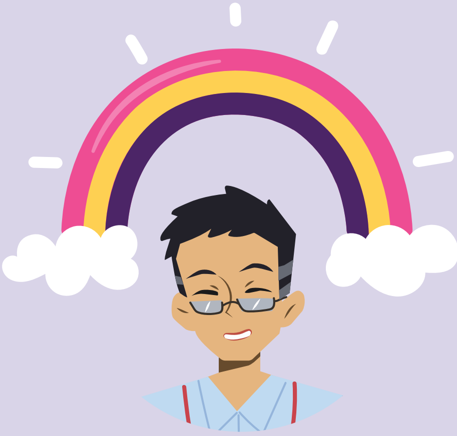

Head out to your nearest park or green space and take in the relaxing surroundings of being outdoors and in nature while also getting your steps in.

Listen to one of your favourite songs! You can choose songs that bring back good memories, lift your spirits, or help you to de-stress and relax.

Think about your values and write them down in your journal, or use self-talk to repeat them to yourself.

For example "I am at peace with life" or
"I deserve to be loved and respected".

Do something kind for someone else like holding the door open, getting someone coffee, or offering to help with a task.

LIFE HACKS: STRESS LESS

MAKE TIME FOR LOVED ONES

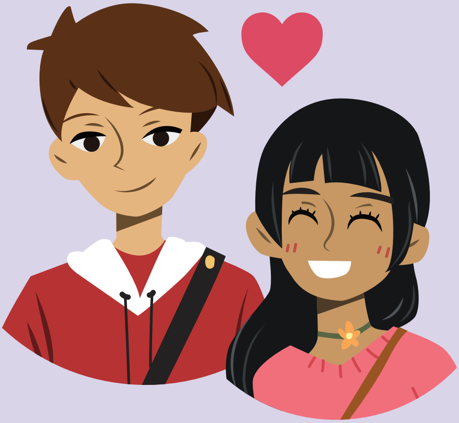

WISH SOMEONE A GOOD DAY

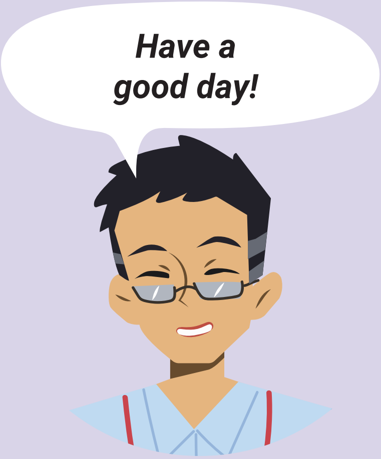

WRITE IT DOWN

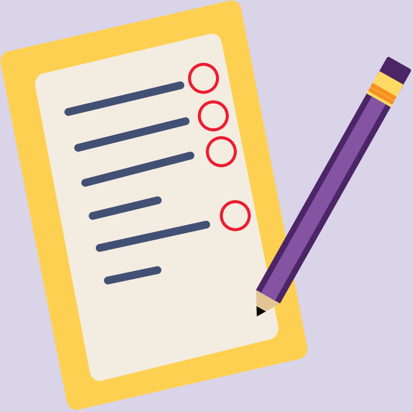

READING BREAK

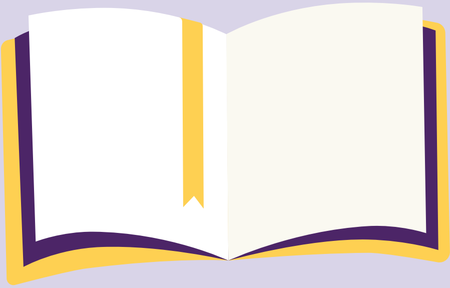

Just initiating a simple greeting can help boost our own happiness and satisfaction in life.

Give a simple "Have a good day!" to someone you care about.

Even if it's just for 10 minutes, take some time to catch up with your friends or family whether it is in-person, or via a text or call.

Pick up a book and take some time to get immersed in another story.

Journaling gives you an outlet to express your deepest emotions in a private space.

Write about your worries, your favourite moment of the day, or a to-do list to plan your next day.

LIFE HACKS: STRESS LESS

MASTER YOUR NOTIFICATIONS

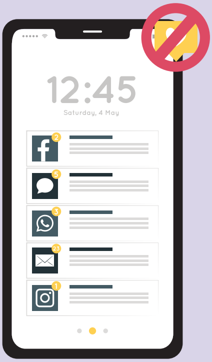

HAPPY MEMORIES

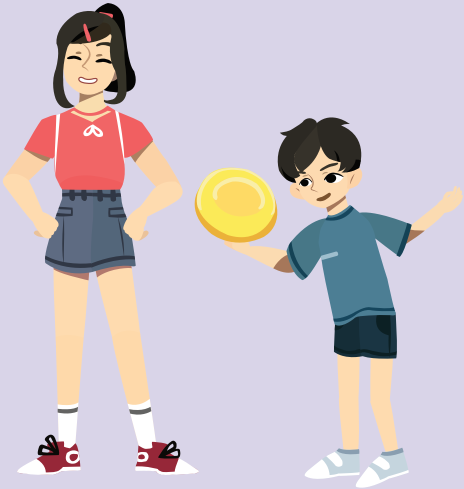

HAVE A LAUGH

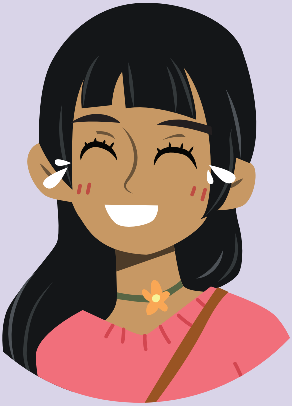

PRACTICE GRATITUDE

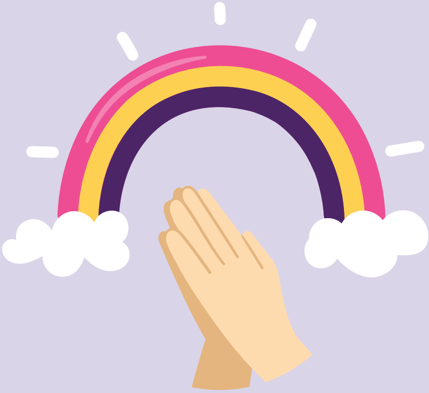

Think back to some happy memories of the past.

Perhaps when you were a child, on holiday, or simply enjoying your favourite food.

Every notification is a potential trigger for distraction.

Reduce distractions from your phone or other devices by turning off unnecessary notifications.

Think about 3 things you are grateful for.

If they involve another person, recognise and thank them whenever possible.

Find some time to add a healthy dose of amusement into your day, such as watching a funny video, joking with those around you or playing with a cute animal.

LIFE HACKS: STRESS LESS

OUT OF SIGHT OUT OF MIND

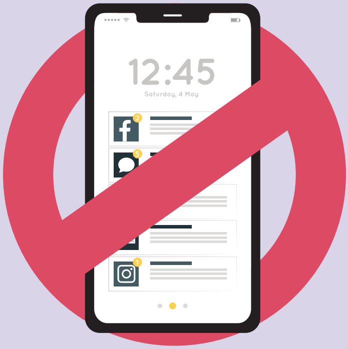

SAY "NO" TO CAFFEINE

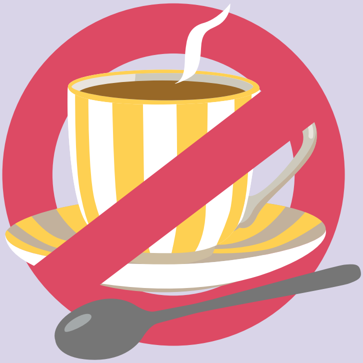

SET YOUR INTENTION

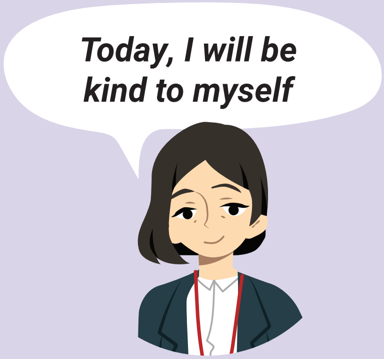

DECLUTTER YOUR SPACE

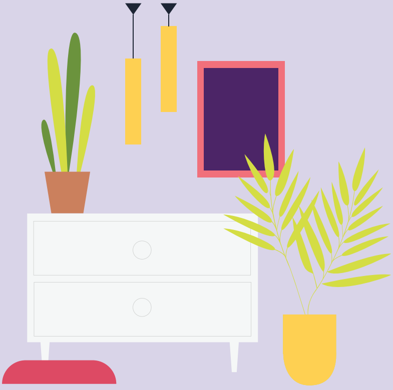

Having a caffeinated drink like coffee, tea or an energy drink in the afternoon may keep you feeling awake and disrupt sleep.

Challenge yourself not to consume anymore caffeinated drinks after 3pm.

Keep your phone out of sight for at least an hour to reduce distractions.

Take 5 minutes to declutter your workspace and clear your mind in the process.

Set your intention for the day.

For example, "Today, I will be kind to myself, eat well, and stay grounded."

LIFE HACKS: MOVE MORE

THE SUNSHINE VITAMIN!

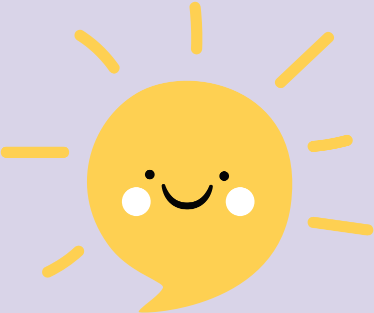

BREAK IT UP

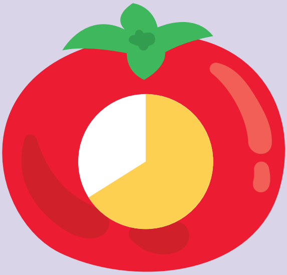

TV EXERCISE

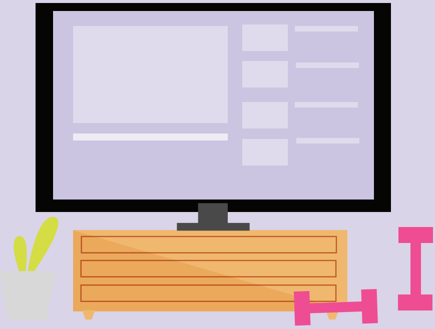

DITCH THE CAR

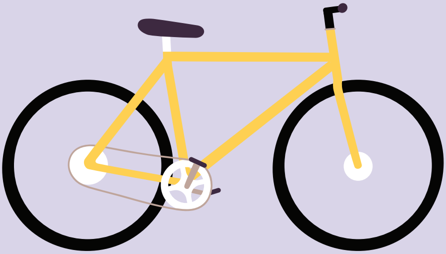

Set a timer to work or study for 30-40 minutes, then stand-up for a 5-minute break.

Go outdoors and enjoy the sunshine for 15 mins.

You'll get your daily dose of vitamin D while getting your steps in!

Instead of getting in a car, take public transport or, even better, walk or cycle to get to where you need to go.

Watch your favourite show while doing some stretches, squats, push-ups or even dancing!

LIFE HACKS: MOVE MORE

Every minute counts!

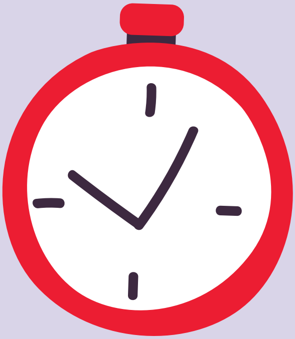

Explore your surroundings

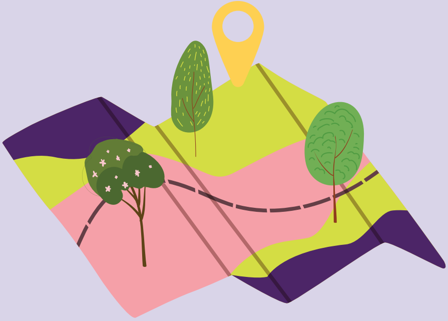

Schedule your exercise

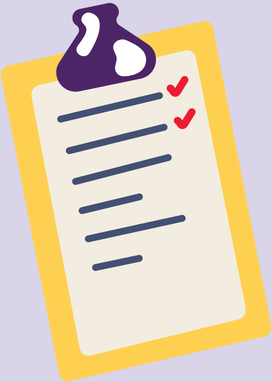

Two birds one stone

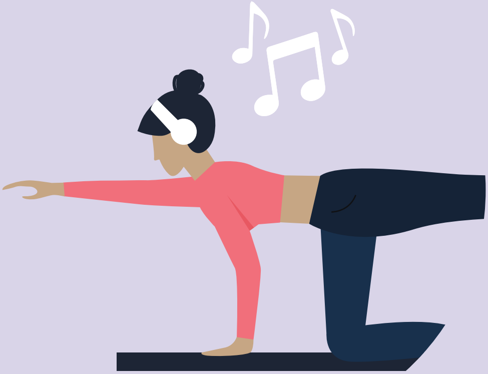

Find a new place in your area to explore on foot or by bike like nearby parks, trails, or interesting buildings.

For just a few minutes, do some walking, stretching, household chores, or anything you like.

Short bursts of activity are better than nothing!

Listen to your favourite music, a podcast or an audiobook, or even watch a movie while exercising!

Schedule some exercise in your calendar for the upcoming week, just like you would schedule a meeting in your calendar.

LIFE HACKS: MOVE MORE

EXERCISE BUDDY

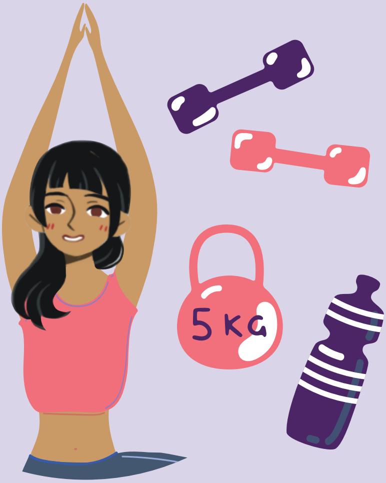

FEEL-GOOD EXERCISE

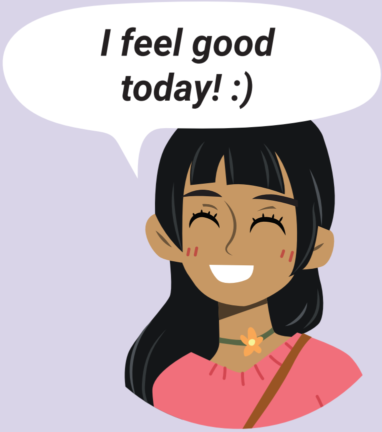

LUNCHTIME ACTIVITY

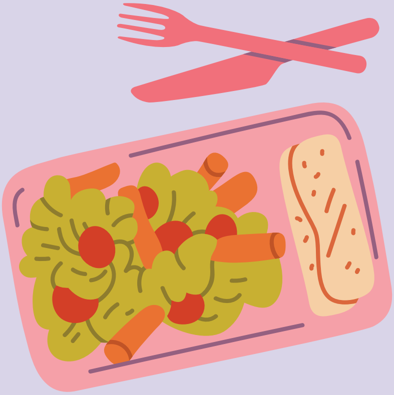

STRETCH TO A CLEARER MIND

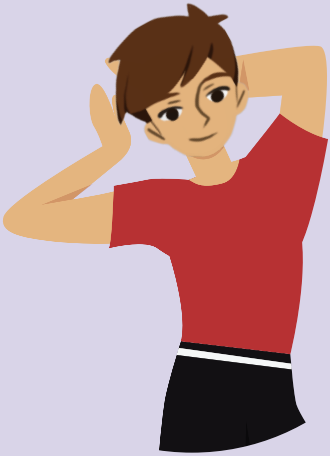

Remind yourself how good it feels after doing some exercise or how proud you are of your regular exercise.

For example: "I feel good when I move" or "I've worked hard to be active".

Find a partner or group to exercise with. You can motivate each other!

Take a couple of minutes to do some simple stretches e.g. reaching as far down to your toes and as high up to the sky/ceiling; shoulder and neck rolls; twisting around the torso.

Sneak in 10-15 mins of physical activity during your lunch break to get your blood pumping and break up prolonged sitting.

LIFE HACKS: MOVE MORE

DON'T JUST WAIT AROUND

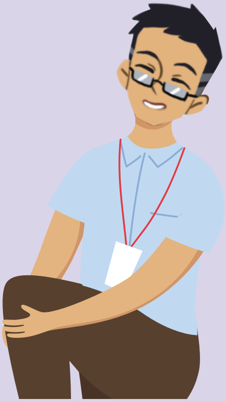

ELEVATE YOURSELF

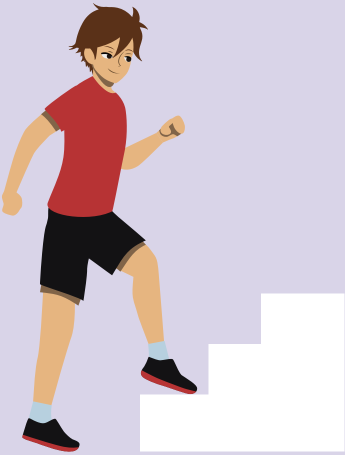

EXERCISE MOTIVATION

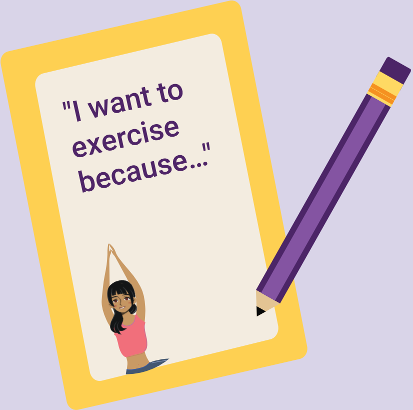

WALK IN PLACE

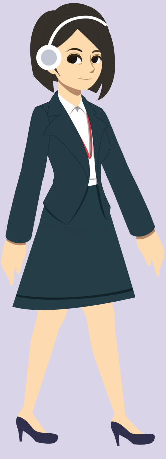

Stair climbing is a whole-body workout that increases strength and fitness, and reduces blood pressure.

Take at least two flights of stairs before using the lift today.

Instead of looking at your phone while waiting for the MRT, for the microwave to ding, or queuing in line, do some toe raises, squats, or stretch your neck and arms.

Do you have a Zoom meeting or phone call today?

Pace or walk in place while you engage in conversation to increase your step count!

Studies show that people are more successful if their exercise motivation comes within.

Write down what motivates you to exercise, for example: "I want to exercise because..."

Slow-paced breathing

Regular breathing typically falls within a range of 12-18 breaths/min.

By setting a time for inhaling and exhaling, we can train ourselves to bring that number down to 5-7 breaths/min, which is, scientifically, just the right number to support the function of body and mind.

Just 5 minutes every day is enough to see a noticeable change, and I realised that through regular practice, I felt more calm, even the people around me noticed I was more zen.

Here's a demonstration of how we can all practise slow-paced breathing. If you'd like to try something more interactive, head over to the LvL^{UP} app where you can try our innovative new slow-paced breathing tool, Breeze.

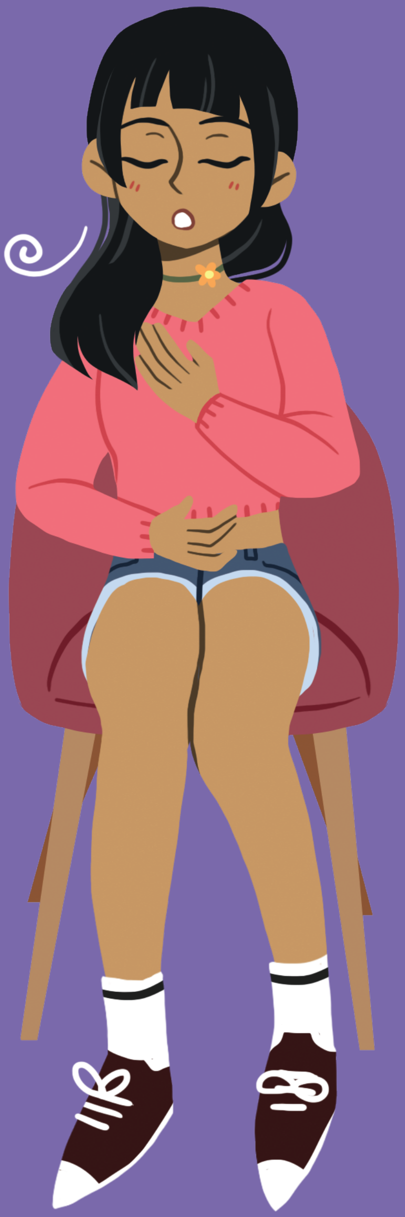

SLOW-PACED BREATHING

Why should I practise slow-paced breathing?

Slow-paced breathing is a natural tranquilliser helping you have a lower heart rate and lower blood pressure, which is a benefit for our health. This exercise is subtle when you first try it but gains in power with repetition and practice.

When and how often should I practise slow-paced breathing?

You can practise slow-paced breathing whenever anything upsetting happens - before you react, whenever you are aware of internal tension, or to help you fall asleep. To receive maximum benefits, make slow-paced breathing a regular practice and do it at least twice a day between 5 to 10 minutes at your own pace and comfort.

How do I practise slow-paced breathing?

Ideally, sit with your back straight, relaxing your neck, shoulders, and upper back. Place a hand on your stomach. You can also practise while standing or laying down if this is more comfortable for you.

Scan QR code to practise slow-paced breathing using our interactive Breeze tool on the LvLUP app.

GET IT ON Google Play

Download on the App Store

SLOW-PACED BREATHING

STEPS

1 Inhale through your nose to a count of 4 and feel the air flow through your body and into your stomach.

2 Hold your breath for a count of 1.

3 Exhale completely through your mouth to a count of 5.

4 This is one breath.
Now inhale again and repeat the cycle three more times for a total of four breaths.

INHALING

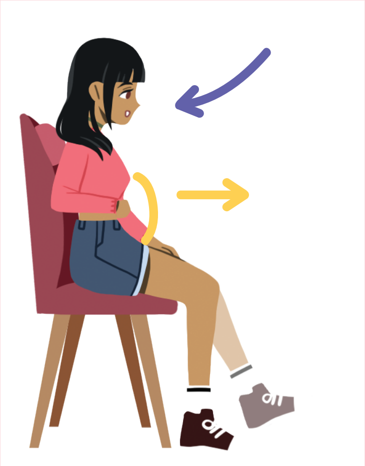

EXHALING

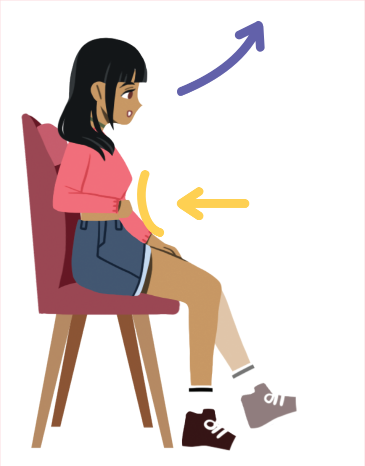

Journal

Journaling gives us our own space to freely and authentically express ourselves.

For me, I found that journaling helps me be more introspective, because I am able to organise and process my thoughts and feelings.

In the next pages, you'll find some helpful templates, as well as some examples of different kinds of journaling which have been proven to produce beneficial effects.

I hope these examples can inspire you, but remember, in the end, your feelings are the most important. Happy writing!

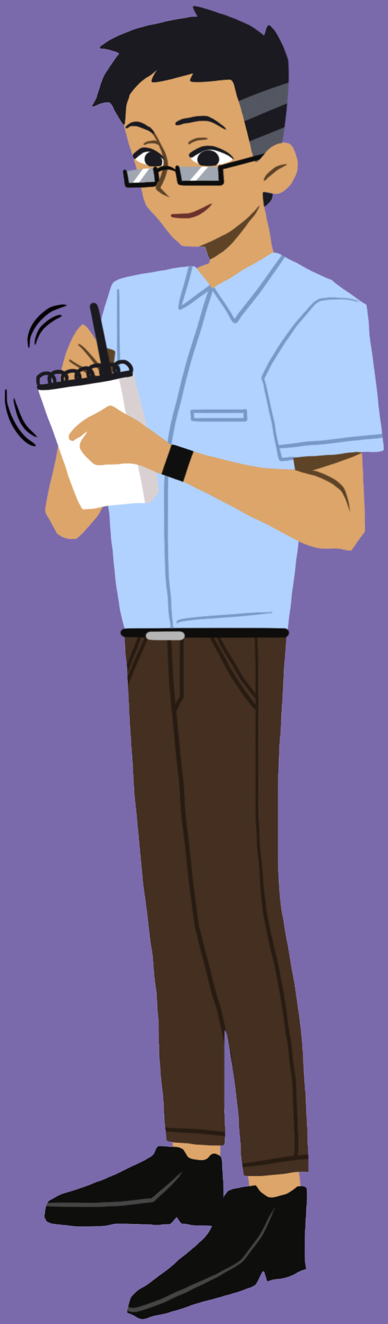

THOUGHTS, FEELINGS, AND BEHAVIOURS

DATE: ____________ **TIME:** ____________

MOOD RATING:

HAPPY 😁 **SAD** ☹ **ANXIOUS** 😰 **ANGRY** 😠

I'm still so shaken by the ride home. It was already raining heavily to begin with and you'd think people would take extra caution, but no. This guy literally came out of nowhere and cut us off!

Thoughts: How can people be so irresponsible on the road? Luckily my taxi driver slammed the brakes just in time or else I don't know what would have happened. The situation was just so out of my hands...

Feelings: I was so scared and I could feel the tension in my neck and shoulders and my heart starting to beat faster. It took me a while to steady my breath once I realised we were okay.

Behaviours: The taxi uncle and I made sure we were both okay and ended up talking and having a really nice conversation. It was nice that even though the situation started off really scary and unpleasant, I ended up getting to connect with a stranger in a way I otherwise wouldn't have.

GRATITUDE JOURNALING

DATE: ____________ **TIME:** ____________

MOOD RATING:

HAPPY **SAD** **ANXIOUS** **ANGRY**

3 things I am grateful for today:

1. *I'm grateful that I got a seat on the MRT this morning*

2. *I'm grateful that I had good music that I enjoy listening to and keeps my spirits up throughout the day*

3. *I'm grateful for the food I ate that gives me energy*

FREE TEXT

DATE: ____________ **TIME:** ____________

MOOD RATING:

HAPPY **SAD** **ANXIOUS** **ANGRY**

Kai was feeling really down about how things were going with his relationship with Alex and seemed at a loss as to what he could do to make the situation better. It was such a mad rush to get everything ready for this product launch, but I couldn't just watch my friend struggling without at least trying to help, even if it's just giving a listening ear. So over our lunch break, I made sure to set aside some time to ask how he was doing.

Kai confided in me about how he'd been feeling and the difficulties he'd been facing with juggling all his commitments. Although I couldn't offer a solution, Kai thanked me for giving him a space where he could vent his frustrations and said he felt better afterwards. I was really happy to hear that my small action was able to help my friend in some way. It made me realise just how little effort it can take to bring positivity into the lives of others, and in turn, ourselves.

DATE: ____________ TIME: ____________

MOOD RATING:

HAPPY SAD ANXIOUS ANGRY

DATE: ____________ TIME: ____________

MOOD RATING:

HAPPY SAD ANXIOUS ANGRY

DATE: ____________ TIME: ____________

MOOD RATING:

HAPPY SAD ANXIOUS ANGRY

DATE: ____________ TIME: ____________

MOOD RATING:

HAPPY SAD ANXIOUS ANGRY

DATE: ____________ TIME: ____________

MOOD RATING:

HAPPY SAD ANXIOUS ANGRY

DATE: ____________ TIME: ____________

MOOD RATING:

HAPPY SAD ANXIOUS ANGRY

DATE: ____________ TIME: ____________

MOOD RATING:

HAPPY SAD ANXIOUS ANGRY

DATE: ______________ **TIME:** ______________

MOOD RATING:

HAPPY SAD ANXIOUS ANGRY

DATE: ______________ TIME: ______________

MOOD RATING:

HAPPY SAD ANXIOUS ANGRY

DATE: ____________ TIME: ____________

MOOD RATING:

HAPPY SAD ANXIOUS ANGRY

DATE: ____________ TIME: ____________

MOOD RATING:

HAPPY SAD ANXIOUS ANGRY

DATE: ____________ TIME: ____________

MOOD RATING:

HAPPY SAD ANXIOUS ANGRY

DATE: ____________ **TIME:** ____________

MOOD RATING:

HAPPY SAD ANXIOUS ANGRY

DATE: ____________ TIME: ____________

MOOD RATING:

HAPPY SAD ANXIOUS ANGRY

DATE: ______________ TIME: ______________

MOOD RATING:

HAPPY SAD ANXIOUS ANGRY

DATE: ____________ TIME: ____________

MOOD RATING:

HAPPY SAD ANXIOUS ANGRY

JOURNAL

A WEEK OF:

MON

TUE

WED

THU

FRI

SAT

SUN

HIGHLIGHTS OF THE WEEK

TO-DO LIST

LvL UP™

JOURNAL

A WEEK OF:

MON

TUE

WED

THU

FRI

SAT

SUN

HIGHLIGHTS OF THE WEEK

TO-DO LIST

JOURNAL

A WEEK OF:

MON

TUE

WED

THU

FRI

SAT

SUN

HIGHLIGHTS OF THE WEEK

TO-DO LIST

JOURNAL

A WEEK OF:

MON

TUE

WED

THU

FRI

SAT

SUN

HIGHLIGHTS OF THE WEEK

TO-DO LIST

LvL UP

JOURNAL

A WEEK OF:

MON

TUE

WED

THU

FRI

SAT

SUN

HIGHLIGHTS OF THE WEEK

TO-DO LIST

LvL UP

JOURNAL

A WEEK OF:

MON

TUE

WED

THU

FRI

SAT

SUN

HIGHLIGHTS OF THE WEEK

TO-DO LIST

JOURNAL

A WEEK OF:

MON

TUE

WED

THU

FRI

SAT

SUN

HIGHLIGHTS OF THE WEEK

TO-DO LIST

JOURNAL

A WEEK OF:

MON

TUE

WED

THU

FRI

SAT

SUN

HIGHLIGHTS OF THE WEEK

TO-DO LIST

JOURNAL

A WEEK OF:

MON

TUE

WED

THU

FRI

SAT

SUN

HIGHLIGHTS OF THE WEEK

TO-DO LIST

JOURNAL

A WEEK OF:

MON

TUE

WED

THU

FRI

SAT

SUN

HIGHLIGHTS OF THE WEEK

TO-DO LIST

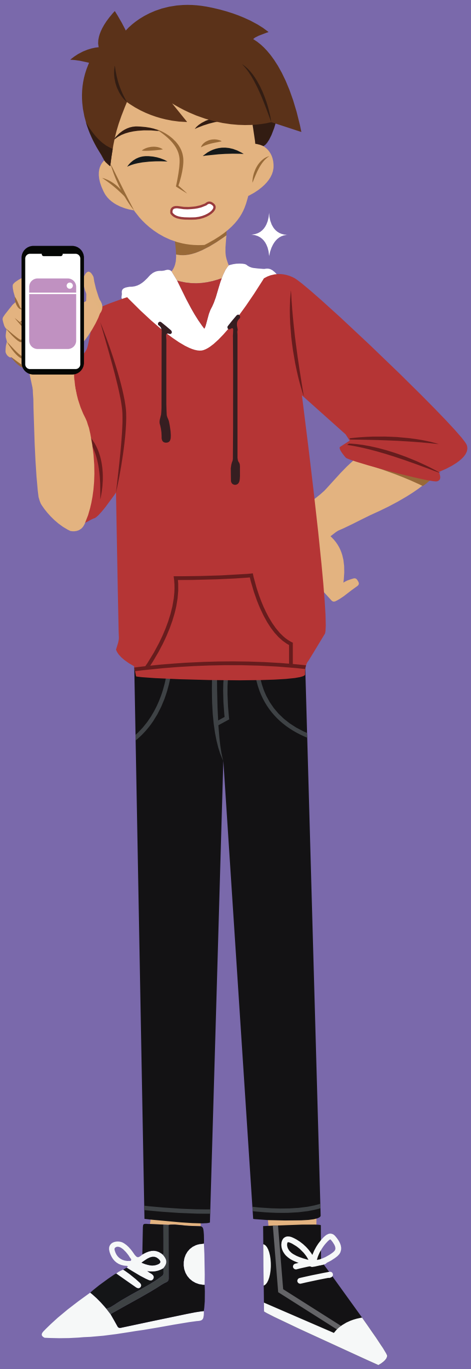

LvLUP APP

As you know, LvLUP also comes in a mobile app form.

There, you will find life hacks, a slow-paced breathing game and an online journal, and also many other exciting things that can't be found in this booklet! Here, let me show you!

This here is the LvLUP cockpit, where you can see everything about your personal LvLUP journey in one place. You can look at all the tasks you need to complete to get to the next level, a history of everything you've accomplished in LvLUP, and easily access any one of the tools.

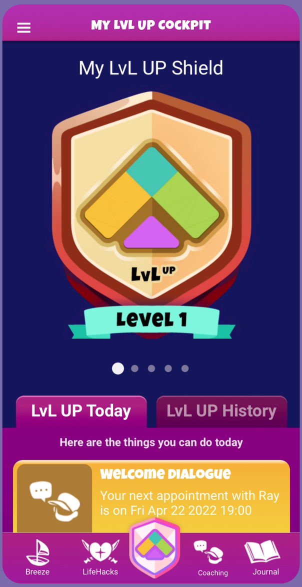

What would LvL^{UP} be without levels?

In the app, you get to work through three levels, each one with new challenges and goals.

Every time a level is completed, you will be rewarded with a new episode about how my friends and I experienced our own LvL^{UP} journeys.

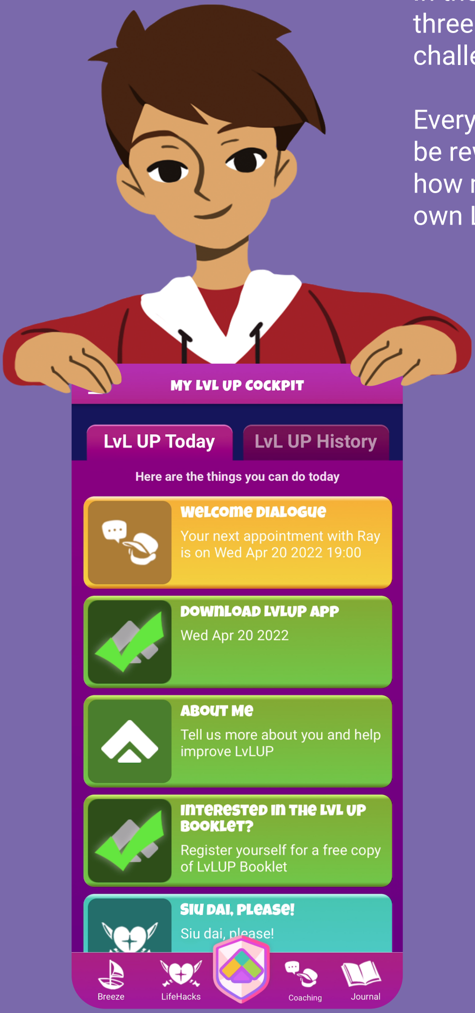

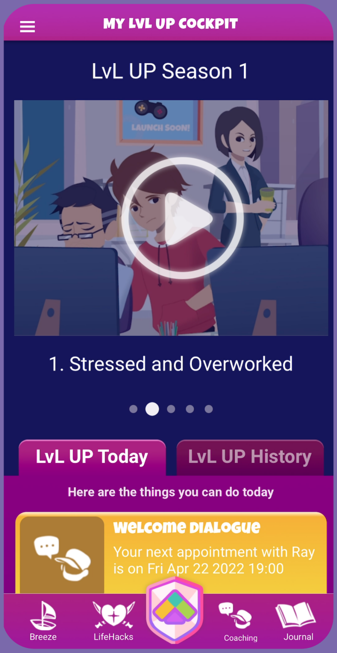

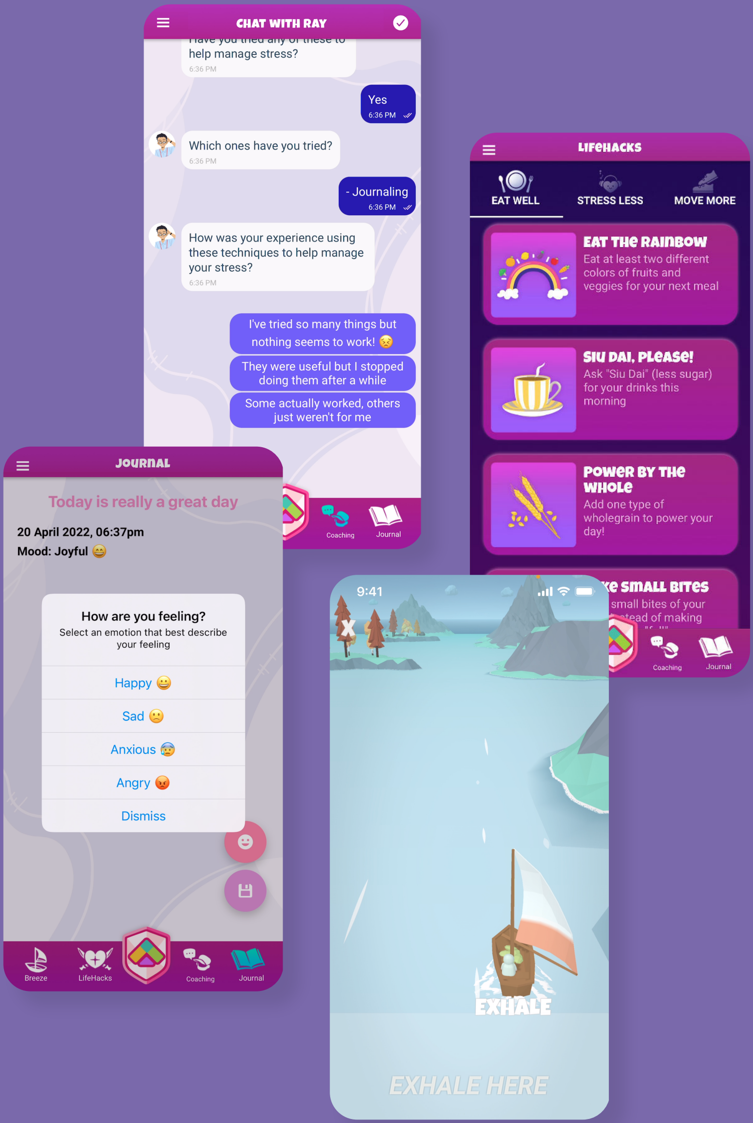

Just look at all these cool features! The LvL^UP app is the perfect complement to this booklet and vice versa. It's all about that balance after all, am I right?

Scan QR code to download the app

GET IT ON Google Play

Download on the App Store

MEET THE TEAM

LvLUP was created by a multidisciplinary team of local and international researchers and scientists from world-renowned institutes like National University of Singapore (NUS), Nanyang Technological University (NTU) and ETH Zurich.

Using the latest cutting edge research in the fields of health behaviour change, medicine, physical activity, diet and nutrition, clinical psychology, and computer science, they have partnered with Lionfish Studios, a local game development studio who helped bring everything you see to life! These next pages represent just some of the 30+ people who have worked together over the past 18 months to bring you LvLUP.

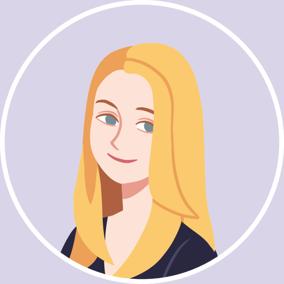

Dr Jacqueline Mair

Behavioural scientist and project leader (diet and physical activity). Loves yoga, sushi, and family adventures.

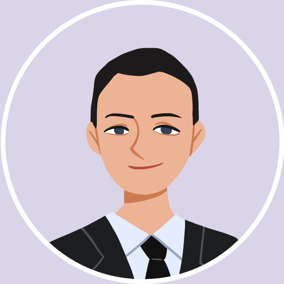

Asst Prof. Tobias Kowatsch

LvLUP creative director and executive producer. Loves tech, movies and biohacking.

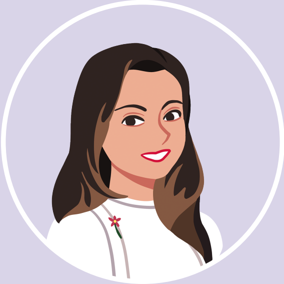

Dr Alicia Salamanca

Clinical psychologist and project leader (mental health). Loves travelling, watching movies, and hiking.

MEET THE TEAM

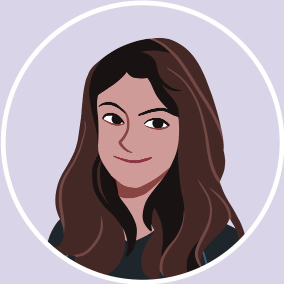

Aishah Alattas

Digital health producer, researcher and leader of the Stress Less pillar. Loves books, chocolate and theatre.

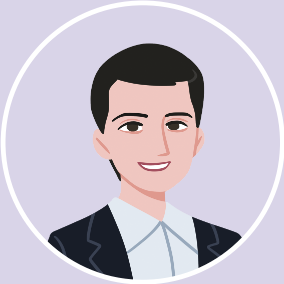

Roman Keller

Researcher and leader of the Move More pillar. Loves cycling, nature and music.

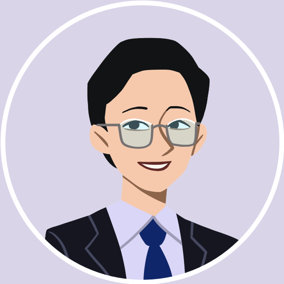

Lim Chang Siang

Software engineer and developer of the LvL^{UP} App. Loves books and code.

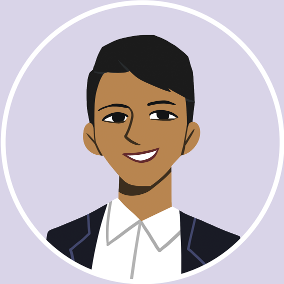

Ahmad Ishqi Jabir

Researcher and leader of the Eat Well pillar. Loves long walks, k-drama and cooking.

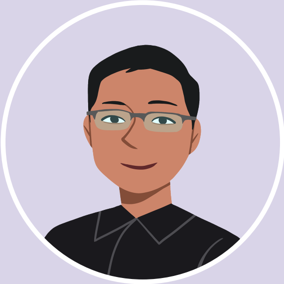

Prof. E Shyong Tai

Doctor interested in diabetes. Loves food, hates exercise, but willing to exercise so that he can eat!

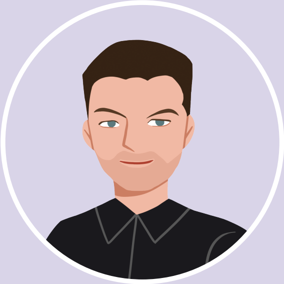

Prof. Florian von Wangenheim

Technology marketing professor. Loves LvL^{UP}, sports and food.

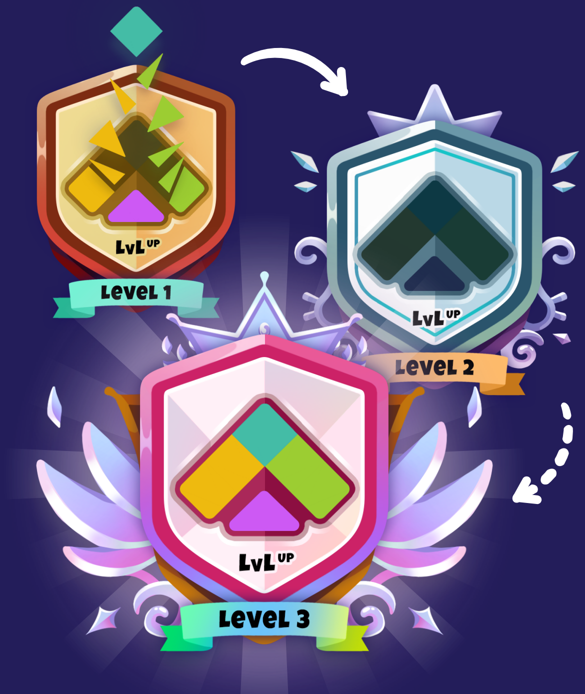

LvL UP YOURSELF AT LVLUP.COM.SG

This research is supported by the National Research Foundation, under its Campus for Research Excellence and Technological Enterprise (CREATE) programme.

ETH Singapore SEC Ltd (UEN 201016935D)
1 Create Way
CREATE Tower #06-01
Singapore 138602

Singapore-ETH Centre is the registered business name of ETH Singapore SEC Ltd.

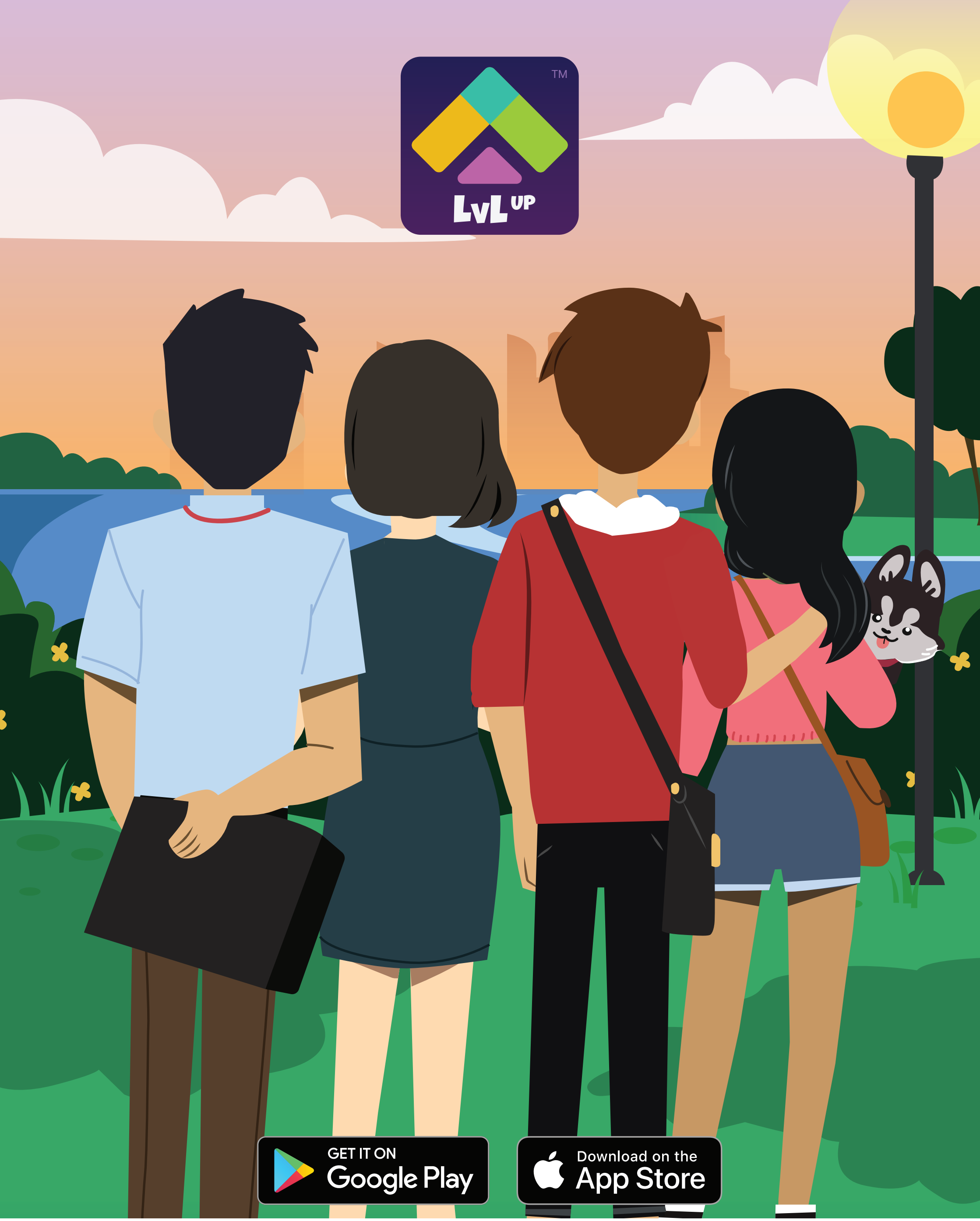

NUS National University of Singapore

LEE KONG CHIAN SCHOOL OF MEDICINE

(SEC) SINGAPORE-ETH CENTRE

ETH zürich

LIONFISH STUDIOS
